# Enterovirus Testing in Hand, Foot, and Mouth Disease and Herpangina: A Highly Sensitive Single-Round VP4–VP2 Reverse-Transcription Polymerase Chain Reaction Assay with a Redesigned Reverse Primer

**DOI:** 10.3390/v18050527

**Published:** 2026-04-30

**Authors:** Tsuguto Fujimoto, Miki Ogi, Kazuhiro Kitakawa, Takako Sano, Yorihiro Nishimura, Kouichi Kitamura, Minami Kikuchi Ueno, Minetaro Arita

**Affiliations:** 1 Department of Fungal Infection, National Institute of Infectious Diseases, Japan Institute for Health Security (JIHS), Tokyo 162-8640, Japan; 2 Infectious Disease Research Division, Hyogo Prefectural Institute of Public Health Science, Kakogawa 675-0003, Japan; miki_ogi@pref.hyogo.lg.jp; 3 Fukushima Prefectural Institute for Public Health, Fukushima 960-8560, Japan; kitakawa_kaduhiro_01@pref.fukushima.lg.jp; 4 Microbiology Division, Kanagawa Prefectural Institute of Public Health, Chigasaki 253-0087, Japan; sano.ipn@pref.kanagawa.lg.jp; 5 Department of Virology II, National Institute of Infectious Diseases, Japan Institute for Health Security (JIHS), Tokyo 208-0011, Japan; nishimura.yo@jihs.go.jp (Y.N.); kitamura.k@jihs.go.jp (K.K.); ueno.mi@jihs.go.jp (M.K.U.); arita.m@jihs.go.jp (M.A.); 6 Department of Diagnostic Testing and Technology Research, National Institute of Infectious Diseases, Japan Institute for Health Security (JIHS), Tokyo 208-0011, Japan

**Keywords:** hand, foot, and mouth disease, herpangina, enterovirus, reverse-transcription polymerase chain reaction

## Abstract

Molecular typing of enteroviruses (EVs) is essential for surveillance of hand, foot, and mouth disease (HFMD). Conventional reverse-transcription polymerase chain reaction (RT-PCR) targeting the VP4–VP2 region can be insufficiently sensitive, reducing the detectability of Enterovirus A (EV-A). We developed a single-round RT-PCR assay using a modified reverse primer design (C3R) for rapid EV detection and genotyping. Sensitivity was evaluated using EV-A71 and poliovirus type 1 reference strains, across 60 EV-positive clinical specimens. The C3R-based assay showed ~1000-fold higher sensitivity for EV-A71 than for conventional assays (limit of detection: 6.6 copies/reaction). The assay detected 98.3% (59/60) of clinical specimens in a single-round format, whereas the conventional assay detected only 45.0% (27/60) and showed a marked decline in detection at higher Ct values. The C3R-based assay maintained complete detection for clinical specimens with Ct values below 40. The majority of the amplified products yielded high-quality sequences suitable for genotyping. This C3R-based RT-PCR overcomes sensitivity limitations of existing protocols and provides reliable genotyping from low-viral-load specimens, supporting its use in routine diagnostics and large-scale HFMD surveillance.

## 1. Introduction

Over 116 types of enteroviruses (EVs) infect humans, although some have unknown pathogenicity. Among them, polioviruses (PVs) and many non-polio enteroviruses (NPEVs) circulate globally [1,2]. The major types that circulate in Japan include Coxsackievirus A (CVA), which belongs to the species *Enterovirus alphacoxsackie* (formerly *Enterovirus A*; EV-A); Coxsackievirus B (CVB) and echoviruses, both classified within *Enterovirus betacoxsackie* (formerly *Enterovirus B*; EV B); Enterovirus D68, a member of *Enterovirus deconjuncti* (formerly *Enterovirus D*; EV D); and EV-A71, which is also included in EV-A [3,4]. EV-A types such as CVA6, CVA10, CVA16, and EV-A71 commonly cause hand, foot, and mouth disease (HFMD), as consistently reported in recent epidemiological studies in Asia and Europe, whereas several CVA (EV-A) types cause herpangina. The EV-B types, including CVB and echoviruses, can also contribute to both [5,6]. Although EV-A71 is recognized as a highly neurotropic virus capable of causing severe neurological complications, including acute flaccid paralysis (AFP), EV-A71 outbreaks in Japan have been reported primarily in association with aseptic meningitis and encephalitis, with AFP cases occurring only sporadically [7]. These diseases predominantly affect children and significantly impact public health [4].

Epidemic patterns shift annually in Japan, where EV-A71 outbreaks are associated with neurological complications such as aseptic meningitis and encephalitis [4,7,8]. Severe cases involving CVA2 and CVA4 have also been reported in the country [9,10], highlighting the importance of timely and accurate typing to support diagnoses, outbreak responses, and surveillance.

Semi-nested reverse-transcription polymerase chain reaction (RT-PCR) of the VP4–VP2 region (hereinafter referred to as conventional VP4–VP2 RT-PCR) has been widely used since the 1990s as a reliable and simple method for detecting and typing EVs [11,12]. Conventional VP4–VP2 RT-PCR, along with VP1-based assays such as consensus-degenerate hybrid oligonucleotide primer PCR (CODEHOP PCR) [13], are commonly used in clinical and surveillance settings. VP1 sequencing remains the gold standard for definitive typing and genotyping. Although nested VP1 assays have high sensitivity, their high costs and long turnaround times also limit their utility for rapid or large-scale screening. Notably, recent evidence has indicated that, in most instances, EV types can be assigned directly from conventional VP4–VP2 RT-PCR products via sequence-based analysis, providing a practical complement to VP1-based typing [12]. Nevertheless, conventional VP4–VP2 RT-PCR can underperform in low-viral-load specimens. In this study, “low-viral-load specimens” refer to clinical samples with pan-enterovirus real-time RT-PCR Ct values ≥30, unless otherwise specified. In one case of EV-A71 infection in a cerebrospinal fluid (CSF) sample, conventional VP4–VP2 RT-PCR based on the EVP4/OL68 primer returned negative results, while both 5′-untranslated region (UTR) nested RT-PCR and real-time PCR assays showed positive detection, underscoring a sensitivity gap under low-titer conditions [14]. In this context, more sensitive VP4–VP2 RT-PCR may serve as a rapid screening tool to triage specimens for downstream VP1 or whole-genome sequencing, and as a complementary fallback test when VP1 fails [15], thereby maintaining the continuity of EV typing in real-world clinical and public health workflows. However, the relatively short length of the VP4–VP2 region may limit resolution and discriminatory power for certain genotypes and recombinant strains, particularly when sequence identity is borderline or intra-type variability is high.

In Japan, conventional VP4–VP2 RT-PCR is incorporated into the official HFMD pathogen-detection manual [16] used by public health laboratories (National Institute of Infectious Diseases, 2023). Accordingly, improving the sensitivity of this widely adopted assay has direct operational relevance for routine diagnostics and national surveillance workflows [12]. Recent evidence indicates that EV types determined from VP4–VP2 sequences show high concordance with VP1-based typing in most comparative evaluations [15].

To address these limitations [14], we designed a novel reverse primer, C3R, for VP4–VP2 RT-PCR to improve its detection of EV-A and other EVs associated with HFMD and herpangina. In contrast to the conventional OL68-1 primer, which was designed based on limited historical sequence data and exhibits 3′-end mismatches with contemporary EV-A lineages, the C3R primer was rationally redesigned to target a more conserved VP2 coding region and to improve primer–template compatibility at the 3′ end.

By focusing on a shorter, more conserved target region, our alternative RT-PCR assay enhances analytical sensitivity while preserving type-level resolution via direct Sanger sequencing, offers a faster turnaround time, and reduces contamination risk when compared with nested protocols. Herein, we report the performance of this optimized assay, referred to as C3R-based RT-PCR, compared with that of conventional OL68-1-based RT-PCR [11,12], using a specimen set enriched in HFMD and herpangina cases.

Given the recurrent surges of HFMD and herpangina in Japan, and the need for rapid identification of neurovirulent EV types [7,17], an assay that reliably types low-viral-load specimens would be highly valuable for both patient management and public health decision-making.

## 2. Materials and Methods

### 2.1. Primers and Probes

**Figure 1:**
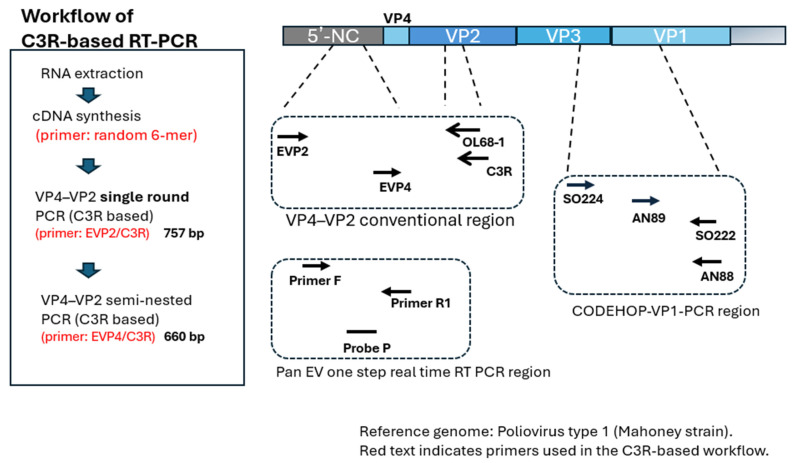
Schematic overview of the C3R-based VP4–VP2 RT-PCR workflow and primer/probe positions across the enterovirus genome. (**Left**) Workflow of the C3R-based VP4–VP2 RT-PCR protocol, including RNA extraction, cDNA synthesis using random hexamers, single-round VP4–VP2 PCR with the EVP2/C3R primer set, and the optional C3R-based semi-nested PCR using EVP4/C3R. (**Right**) Genomic locations of primers and probes used in the pan-enterovirus one-step RT-qPCR [19], conventional VP4–VP2 RT-PCR, semi-nested VP4–VP2 PCR [12], and VP1 CODEHOP assays [13]. The primer and probe binding sites are mapped across the 5′-NC, VP4, VP2, VP3, and VP1 regions to illustrate the target regions for each assay.

All primers and probes are listed in Table 1, and their corresponding genomic binding sites are shown schematically in Figure 1. For the VP4–VP2 single-round and semi-nested PCR assays, the forward primers EVP2 [18] and EVP4 [12] and the reverse primers OL68-1 [11] and C3R were used (the latter being designed by our team to target a conserved VP2 coding region).

For VP1 CODEHOP PCR [13], SO224/SO222 primers were used in the first round, followed by AN89/AN88 ones in the second round.

Pan-EV real-time RT-PCR (i.e., the reference assay) was performed using Primers F and R1, together with the corresponding probe, according to a previously described protocol [19].

### 2.2. Assessment of Analytical Sensitivity Using Viral Isolates

Analytical sensitivity was evaluated using two reference isolates: EV-A71 (Nagoya; GenBank accession no.: AB482183.1), as a representative EV-A strain; and poliovirus type 1 (PV1; Sabin 1; GenBank accession no.: AY184219), as a representative non-EV-A strain. Viral RNA extracted from each isolate was serially diluted and tested side-by-side to compare the detection limits of the C3R-based single-round VP4–VP2 RT-PCR assay and the conventional OL68-1-based single-round VP4–VP2 RT-PCR assay. In this study, “single-round” denotes a non-nested RT-PCR workflow.

Viral RNA was extracted from virus-cultured cells using the Zymo Quick Viral RNA Kit (Zymo Research, Irvine, CA, USA) according to the manufacturer’s instructions. RNA stocks were serially diluted ten-fold (10^−1^–10^−10^), reverse-transcribed using the PrimeScript RT reagent Kit (Takara Bio Inc., Kusatsu, Japan), and subsequently subjected to first- and second-round (semi-nested) PCR using the EmeraldAmp PCR Master Mix (Takara Bio).

Quantification of viral RNA copy numbers was performed by real-time RT-PCR as previously described by Ueno et al. [20]. Quantified viral RNA prepared from the PV1 (Mahoney) strain was used to generate standard curves spanning 10^7^ to 10^2^ copies per reaction, demonstrating linear amplification over this range. Real-time RT-PCR was performed using the One Step SYBR PrimeScript PLUS RT-PCR Kit (Takara Bio) on an Applied Biosystems 7500 Fast Real-Time PCR System(Thermo Fisher Scientific, Waltham, MA, USA), with Ct values obtained after 45 amplification cycles.

The number of viral genome copies per PCR reaction was calculated according to the following formula:

Copies per reaction = C_stock × V_input × D × 1/2

where C_stock is the RNA stock concentration (copies/µL), V_input is the volume equivalent of cDNA input per reaction, and D is the dilution factor. The RNA stock quantified by real-time RT-PCR was diluted by a factor of one-half during the reverse transcription step described above. Based on the quantified copy number of the original RNA stock, the copy numbers of the serially diluted samples used for analytical sensitivity testing were estimated from the corresponding dilution factors. The copy number of the diluted reaction mixtures was not directly re-quantified by real-time RT-PCR at each dilution endpoint.

The limit of detection (LOD) was defined operationally as the lowest RNA dilution at which a specific amplicon could be reproducibly detected in the second-round PCR under the experimental conditions described above, as confirmed in at least two independent experiments.

### 2.3. Clinical Specimens

We retrospectively analyzed 60 clinical specimens collected in Japan between 2011 and 2022 from patients with suspected HFMD or herpangina. Specimens were obtained from two prefectural public health institutes participating in routine HFMD and herpangina surveillance in Japan. VP4–VP2 PCR assays and subsequent sequencing were performed at the two participating laboratories using an identical standardized protocol. Interpretation criteria for gel electrophoresis, positivity calls, and sequencing outcomes were harmonized in advance and applied uniformly across sites. The specimens were originally collected at sentinel pediatric clinics as part of the national infectious disease surveillance program mandated by the Infectious Diseases Control Law. Specimens were included if they tested EV-positive by the reference 5′-NC real-time RT-PCR [19] assay described above.

The specimens included oropharyngeal swabs (*n* = 45), saliva (*n* = 8), stool (*n* = 3), nasal secretions (*n* = 2), nasopharyngeal swab (*n* = 1), and cerebrospinal fluid (*n* = 1).

Co-detection of rhinovirus (RV) by multiplex or RV-specific assays performed at the submitting laboratories was recorded but not used for eligibility determination (i.e., the samples were included irrespective of RV status). Rhinovirus detection was performed independently at the submitting laboratories and was not run in parallel as an internal control in the VP4–VP2 RT-PCR assays.

Viral RNA was extracted from 140 µL of each clinical specimen using a QIAamp Viral RNA Mini Kit (Qiagen, Hilden, Germany) and eluted into 60 µL of Buffer AVE (RNase-free elution buffer). Carrier RNA was not used in this study. The Easy Dilution for real-time PCR kit (Takara Bio) was used as the diluent to make the serial dilutions used for the assay’s sensitivity testing.

All complementary DNA (cDNA) samples were synthesized using a PrimeScript™ RT reagent Kit (Takara Bio). All downstream molecular assays (VP4–VP2 single-round PCR, VP4–VP2 semi-nested PCR, and VP1 CODEHOP PCR, when performed) were carried out using the same cDNA stock prepared from each clinical specimen and stored under standardized conditions.

Each 20 µL reaction contained 4 µL of 5× PrimeScript buffer, 4 µL of Random 6-mer primer (100 µM), 1.0 µL of PrimeScript RT Enzyme Mix I, 1.0 µL of nuclease-free water, and 10 µL of RNA. Incubation was performed at 37 °C for 15 min, followed by enzyme inactivation at 85 °C for 5 s.

### 2.4. Reference Assay for Defining EV Positivity in Clinical Specimens

Inclusion as an EV-positive specimen was determined using a pan-EV one-step real-time RT-PCR targeting the 5′ non-coding (5′ NC/5′ UTR) region (primer sequences presented in Table 1) [19]. This real-time assay served as an eligibility-defining reference test for the subsequent cycle threshold (Ct)-based stratification.

For reactions in which a sigmoid amplification curve was observed but the fluorescence did not cross the assay-defined threshold, the reaction was not classified as negative for analytical purposes. Instead, a conventional Ct value of 45 was assigned [21], and the data were retained in all Ct-based summaries and binning procedures.

### 2.5. Pan-EV One-Step Real-Time RT-PCR

Pan-enterovirus real-time RT-PCR [18] was performed in a 20 µL reaction mixture using the One Step PrimeScript III RT-qPCR Mix with UNG (Takara Bio). Each reaction contained 10 µL of the 2× reaction mix, 1.0 µL of Primer F (10 µM), 1.0 µL of Primer R1 (10 µM), 0.2 µL of Probe P (10 µM), 0.4 µL of ROX Reference Dye II (50×), 2.0 µL of extracted RNA, and 5.6 µL of RNase-free H_2_O to reach a final volume of 20 µL.

Real-time RT-PCR was conducted on an ABI QuantStudio 3 instrument (Thermo Fisher Scientific, Waltham, MA, USA). The thermocycling conditions were as follows: 25 °C for 10 min, 52 °C for 5 min, and 95 °C for 10 s, followed by 45 cycles of 95 °C for 5 s and 58 °C for 30 s, with fluorescence detection in the FAM channel.

This assay served as the eligibility-defining reference test in this study, and its Ct values were recorded for downstream stratified analyses.

### 2.6. VP4–VP2 Single-Round PCR (C3R-Based and Conventional OL68-1-Based)

Amplification of the enterovirus VP4–VP2 region was performed using a single-round PCR with EmeraldAmp PCR Master Mix (Takara Bio). To evaluate primer performance, the forward primer EVP2 [18] was paired with either the reverse primer C3R (this study) or OL68-1 [11], and results obtained with the two reverse primer sets were compared. For a head-to-head comparison of reverse primers, C3R- and OL68-1-based VP4–VP2 PCRs were performed in parallel using the same cDNA preparation derived from each clinical specimen. For each specimen, identical reaction conditions, reagent lots, primer concentrations, and thermal cycling programs were used, and reactions were run concurrently in a single PCR run without sample splitting. No specimens were selectively reprocessed based on amplification outcomes.

Each 25 µL reaction contained the 2× EmeraldAmp PCR Master Mix at a final 1× concentration, 0.5 µL of EVP2 (50 µM), 0.5 µL of either C3R or OL68-1 (50 µM), 21 µL of nuclease-free water, and 3 µL of cDNA.

Thermocycling was performed as follows [12,16]: 95 °C for 5 min; 40 cycles of 95 °C for 30 s, 55 °C for 30 s, and 72 °C for 45 s; followed by a final extension at 72 °C for 5 min. PCR products were analyzed by agarose gel electrophoresis.

### 2.7. VP4–VP2 Semi-Nested PCR

The 2nd PCR was performed as a conventional (non-real-time) semi-nested reaction using EmeraldAmp PCR Master Mix (Takara Bio). The 1st-round products served as templates as follows:

1st PCR EVP2/C3R → 2nd PCR EVP4/C3R

1st PCR EVP2/OL68-1 → 2nd PCR EVP4/OL68-1

For each reaction (25 µL total volume), the mixture contained the 2× EmeraldAmp PCR Master Mix at a final 1× concentration, 0.5 µL of EVP4 [12] (50 µM), 0.5 µL of the appropriate reverse primer (C3R or OL68-1; 50 µM), 21 µL of nuclease-free water, and 3 µL of the 1st-round PCR product.

Thermal cycling conditions were: 95 °C for 5 min; 35 cycles of 95 °C for 30 s, 55 °C for 30 s, and 72 °C for 45 s; followed by a final extension at 72 °C for 5 min. Amplicons were resolved by agarose gel electrophoresis. Primer sequences are provided in Table 1.

### 2.8. VP1 CODEHOP PCR [13]

For this assay, the first round (50 µL total volume) was performed using SO224/SO222 primers and cDNA with the 2× EmeraldAmp PCR Master Mix (Takara Bio) at a final 1× concentration. The thermocycling conditions were: 95 °C for 5 min; 35 cycles of 95 °C for 30 s, 42 °C for 30 s, and 72 °C for 30 s; followed by a final extension at 72 °C for 5 min.

The second round (50 µL total volume) was performed using AN89/AN88 primers with the 2× EmeraldAmp PCR Master Mix (Takara Bio) at a final 1× concentration, under the following conditions: 95 °C for 5 min; 35 cycles of 95 °C for 30 s, 60 °C for 30 s, and 72 °C for 30 s; followed by a final extension at 72 °C for 5 min.

### 2.9. Gel Electrophoresis

Amplicons from all assays (including VP4–VP2 RT-PCR rounds 1 and 2) were separated on 1.5% agarose gels.

For routine visualization, agarose gels containing pre-stained nucleic acid dyes were used.

When amplicon sizes were unclear or when additional confirmation was required, selected samples were re-run and visualized after electrophoresis using post-staining with nucleic acid gel stains (MIDORI Green Advance; NIPPON Genetics EUROPE GmbH, Düren, Germany, or GelRed Nucleic Acid Gel Stain; Biotium, Inc., Fremont, CA, USA).

The choice of staining method was limited to visualization purposes and did not affect the interpretation of band presence or analytical sensitivity.

Samples were considered positive when a band was observed at the assay-specific target size.

For the VP4–VP2 assays, the expected amplicon sizes were approximately 750 bp for the first round and 650 bp for the second (semi-nested) round.

Rhinovirus amplicons were distinguished by their characteristically shorter size (approximately 100 bp smaller).

Negative and positive controls were processed in parallel.

Amplicon sizes were assessed by comparison with DNA molecular weight markers, including ExcelBand 100 bp DNA Ladder (DM2100; SMOBIO Technology, Inc., Hsinchu City, Taiwan) and, where indicated, 100 bp H3 DNA Ladder RTU (BRSD003R500; GeneDireX, Inc., Taoyuan City, Taiwan).

**Figure 2:**
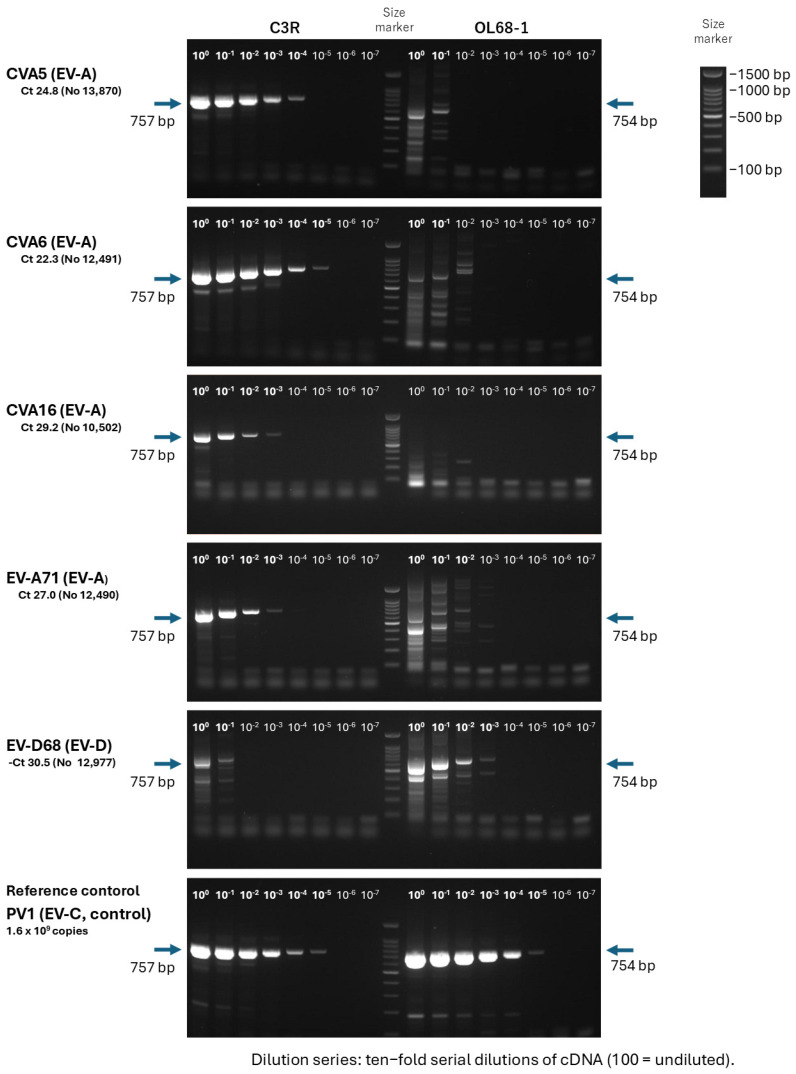
Representative agarose gel electrophoresis demonstrating analytical sensitivity and clinical amplification performance of the C3R-based single-round VP4–VP2 RT-PCR compared with the conventional OL68-1-based assay. PCR was performed using cDNA synthesized from viral RNA extracted from reference isolates or clinical specimens. M, DNA molecular weight marker (100 bp ladder); major size markers (100, 500, 1000, and 1500 bp) are indicated. The expected amplicon sizes are approximately 750 bp for the first-round PCR. The EV-A71 bands shown in this figure are derived from an EV-A71-positive clinical specimen, not from the reference isolate. Arrows indicate the expected VP4–VP2 amplicon bands.

Representative size references (100, 500, 1000, and 1500 bp) are indicated in Figure 2 and Figure S1.

### 2.10. Sanger Sequencing and EV Typing

The PCR products were either enzymatically cleaned using ExoSAP-IT (Applied Biosystems, Thermo Fisher Scientific, Waltham, MA, USA) or gel-purified using the QIAquick Gel Extraction Kit (Qiagen), and subsequently sequenced in both directions using amplification primers. Their chromatograms were visually inspected for quality. Low-quality 5′ and 3′ ends were trimmed, and forward/reverse reads were assembled into a consensus sequence. Identity scores were calculated using a defined alignment window spanning the VP4–VP2 amino acid region after trimming low-quality termini to ensure reproducibility. Ambiguous bases were resolved by reexamining the electropherograms or through repeat sequencing when necessary.

EV typing was performed based on the VP4–VP2 region, following the criteria and thresholds established by Kitamura and Arita [15]. Type assignment was determined based on the percentage of amino acid identity relative to the reference prototype strains. A 95% identity threshold was used as the primary discriminator, while accounting for established exceptions (e.g., PV1–PV2, CVA6, and specific EV-C types). In cases where the identity values were borderline or fell within known exception ranges, the final assignment was supported by VP1 sequencing and phylogenetic analyses [13]. Borderline identity results generally corresponded to amino acid identity values of approximately 92–96%.

Multiple sequence alignment and phylogenetic analyses were performed according to the standard procedures described in the Japanese HFMD pathogen detection manual. Briefly, VP4–VP2 nucleotide sequences were aligned using MEGA software (MEGA v10.1.8), followed by manual inspection and adjustment to ensure correct positional homology. Phylogenetic trees were constructed using the neighbor-joining method based on pairwise genetic distances, and the robustness of tree topology was assessed by bootstrap analysis [16].

### 2.11. In Silico Primer Coverage and 3′-End-Weighted Suitability Scoring (C3R vs. OL68-1)

Primer–template alignments and in silico fitness scores for C3R and OL68-1 across 100 representative enterovirus genotypes are provided in Supplementary Table S2.

#### 2.11.1. Panel and Alignment

The reverse primers C3R (i.e., the experimental primers) and OL68-1 (i.e., the control primers) were assessed against a curated panel of 100 representative EV genotypes (National Center for Biotechnology Information (NCBI) taxonomy ID: 12059) spanning EV-A, EV-B, EV-C, and EV-D (accession numbers listed in the Supplementary Table S2). The in silico panel comprised 100 representative genotypes, including 19 EV-A, 57 EV-B, 20 EV-C, and 4 EV-D types. For each genotype, we extracted the 20 nt reverse primer-binding segment in reverse-complement orientation and aligned the primers accordingly, emphasizing extension from the primer 3′ end.

#### 2.11.2. International Union of Pure and Applied Chemistry (IUPAC) Handling

Ambiguous nucleotide symbols were interpreted according to the standard International Union of Pure and Applied Chemistry (IUPAC) definitions [22].

For each sequence position *i*, primer and template nucleotides were considered concordant when the intersection of their IUPAC-defined nucleotide sets was non-empty; otherwise, the position was scored as a mismatch.

Primer-side “N” was treated as universally permissive and therefore always scored as a match, whereas missing or blank template positions were treated as mismatches.

#### 2.11.3. Primer–Template Fit Score Calculation

Primer–template suitability scores were calculated using the IUPAC-based matching rules described above, combined with a 3′-end-weighted positional scoring model.

A 20 nt window at the 3′ end was evaluated using position-specific weights (positions 1–12 = 0.2; 13–16 = 0.5; 17–20 = 1.0).

The mismatch indicator $d_{i}$ was defined as 0 for matches and 1 for mismatches, and the final score was normalized to a 0–100 scale as follows:

$$\mathrm{Score}=100\times \left(1-\frac{\sum_{i}w_{i}d_{i}}{\sum_{i}w_{i}}\right).$$

For pre-aligned primer–template pairs, the full alignment length was evaluated rather than restricting the analysis to the rightmost 20 nt.

The web-based scoring tool developed for this study is openly available at: https://fujimoto-t.github.io/primer-score/primer-fit-score-en.html (accessed on 4 March 2026).

#### 2.11.4. Outputs and Interpretation

For each primer × genotype, the final score was computed and summarized by species and genotype (full per-genotype tables presented in the Supplementary Table S2). As operational heuristics for single-round PCR, scores were interpreted as 90–100 (near-perfect 3′-proximal match; high likelihood of amplification), 70–89 (generally acceptable; review potential 3′-proximal issues and consider optimization), 50–69 (suboptimal; potentially reduced efficiency), and <50 (poor; amplification likely impaired). These thresholds reflect established evidence that primer–template mismatches, particularly at or near the 3′ end, disproportionately impair priming and extension, thereby affecting qPCR sensitivity and quantification [23,24]. Accordingly, we prioritized 3′-end integrity over global identity, consistent with common primer-design guidance and reporting standards [19].

## 3. Results

### 3.1. In Silico Evaluation of Reverse Primers (Summary)

To contextualize the wet-laboratory results, we first summarized the in silico evaluation of the reverse primers. Using 100 representative EV genotypes, we assessed 3′-end compatibility of the redesigned C3R and conventional OL68-1 primers. C3R maintains consistently high 3′-end fitness across EV-A, EV-B, EV-C, and EV-D, with only a few moderate drops in EV-B and EV-C. This pattern suggests broadly reliable single-round amplification, particularly in EV-A and a broad subset of EV-B. In contrast, OL68-1 exhibits multiple 3′-proximal mismatches in EV-A and scattered reductions in EV-B and EV-C, consistent with its reduced empirical sensitivity observed later in wet-lab validation.

### 3.2. Analytical Sensitivity with Reference Strains

Using RNA from reference isolates, the analytical sensitivity of the VP4–VP2 RT-PCR assays was evaluated for EV-A71 (Nagoya strain, sub-genogroup B) and PV1 (Sabin 1). The C3R-based VP4–VP2 RT-PCR assay demonstrated higher analytical sensitivity for EV-A71 than for the conventional OL68-1-based assay.

For EV-A71, the limit of detection (LOD) of the C3R-based assay was 66 copies per reaction in the single-round RT-PCR and 6.6 copies per reaction in the second-round (semi-nested) RT-PCR. In contrast, the OL68-1-based VP4–VP2 RT-PCR did not yield a detectable amplicon at or above 6.6 × 10^4^ copies per reaction in the single-round RT-PCR, and required at least 6600 copies per reaction to produce a positive band in the second-round RT-PCR. For PV1 (Sabin 1), both assays showed comparable analytical sensitivity, with a limit of detection of 24 copies per reaction in the second-round RT-PCR.

All analytical sensitivity results obtained with reference isolates were reproducible in at least two independent experiments.

### 3.3. Clinical Performance in HFMD/Herpangina Cohort

Representative agarose gel electrophoresis images summarizing the amplification performance of the C3R- and OL68-1-based VP4–VP2 RT-PCR assays across a range of viral loads, using serial ten-fold dilutions of RNA from EV-positive clinical specimens and reference isolates, are shown in Figure 2.

Across 60 EV/RV-positive specimens (oropharyngeal swabs, *n* = 45; saliva, *n* = 8; stool, *n* = 3; nasal secretions, *n* = 2; nasopharyngeal swab, *n* = 1; cerebrospinal fluid, *n* = 1), Ct values ranged from 22.17 to 45.00 (median, 30.64) (Supplementary Table S1).

The C3R-based single-round VP4–VP2 RT-PCR detected 59 of 60 clinical specimens (98.3%), whereas the conventional OL68-1-based assay detected 27 of 60 specimens (45.0%). Pairwise comparison of results for the same specimen set showed 27 specimens positive by both assays, 32 specimens positive only by the C3R-based assay, no specimens positive only by the conventional assay, and one specimen negative by both assays. McNemar’s exact test demonstrated a significant superiority of the C3R-based assay (*p* = 1.16 × 10^−10^). The overall detection rate of the C3R-based single-round assay was 98.3% (59/60; exact 95% confidence interval, 91.1–99.96%), whereas that of the conventional assay was 45.0% (27/60).

The extra band observed in the single-round PCR using OL68-1 likely represents nonspecific amplification.

When stratified by Ct values, specimens positive by the conventional VP4–VP2 RT-PCR had a lower median Ct (higher viral load) of 27.89 (*n* = 27), whereas specimens negative by the conventional assay had a higher median Ct of 31.66 (*n* = 33) (Mann–Whitney U test, *p* = 2.78 × 10^−4^), consistent with reduced sensitivity at low template abundance. Among the specimens assigned a Ct value of 45 by convention, one specimen was classified as negative by both assays, whereas the remaining Ct = 45 specimen yielded detectable VP4–VP2 amplicons.

In contrast, the C3R-based RT-PCR showed high detection rates across a broad Ct range, including high-Ct specimens. Reactions showing sigmoid amplification curves that did not cross the assay-defined threshold were assigned a Ct value of 45 by convention and retained for analysis, as described in Section 2.

**Figure 3:**
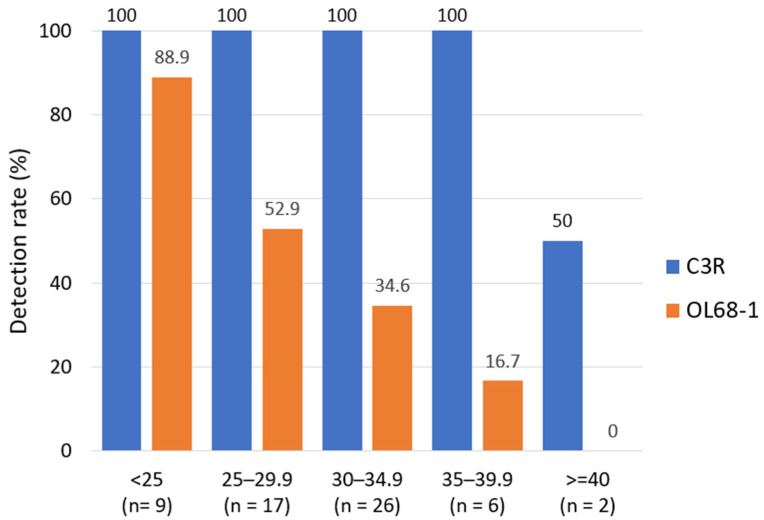
Detection rate of C3R-based and conventional OL68-1-based VP4–VP2 RT-PCR stratified by Ct values obtained from pan-enterovirus real-time RT-PCR. Clinical specimens were stratified into predefined Ct bands (<25, 25–29.9, 30–34.9, 35–39.9, and ≥40). Ct = 45 was assigned by convention to reactions showing sigmoid amplification curves that did not cross the threshold. Numbers below each bar indicate the number of specimens in each Ct category.

These Ct-stratified trends are summarized in Figure 3, which shows that the C3R-based assay maintains detection across Ct strata, in contrast to a sharp decline in detection by the conventional assay beyond Ct 30. Exact 95% confidence intervals (Clopper–Pearson) for Ct-stratified detection rates are provided in Supplementary Table S1.

Consistent with the detection rates shown in Figure 3, VP4–VP2 amplicons obtained from high-Ct specimens were sufficient for direct Sanger sequencing and subsequent molecular typing. Taken together, the visual assessment shown in Supplementary Figure S1 and the corresponding sequencing outcomes indicate that the C3R-based VP4–VP2 RT-PCR frequently yields amplicons suitable for downstream Sanger sequencing from low-viral-load clinical specimens. At the same time, these results also highlight that specimens at the extreme high-Ct range (e.g., Ct = 45) may require nested amplification to obtain interpretable sequence data.

VP4 amino acid identity values were calculated for all 43 clinical specimens for which VP4–VP2 sequencing was performed at one of the two participating laboratories, and the results were compared with four type-specific reference strains (EV-A71, CVA6, CVA10, and CVA16).

Representative results are summarized in Supplementary Table S3. Notably, VP4 amino acid sequences of all EV-A71 specimens were identical to the EV-A71 Nagoya reference strain (146/146 amino acids; 100% identity). In contrast, CVA6 specimens showed substantially lower VP4 amino acid identity values (approximately 86%), whereas CVA10 and CVA16 specimens consistently exhibited high identity values (approximately 98%).

Detection rates of the C3R-based RT-PCR (blue) and the conventional OL68-1-based VP4–VP2 RT-PCR (orange) across predefined Ct strata are shown in Figure 3. The conventional assay demonstrated a pronounced decline in positivity beyond Ct 30, whereas the C3R-based assay maintained high detection across Ct strata, including specimens with Ct values ≥ 40 (Ct = 45 assigned by convention for threshold-negative sigmoid reactions). Sample numbers in each Ct category are indicated in Figure 3.

When grouped by EV species, C3R-based RT-PCR detected EV-A (52/53, 98.3%), EV-B (6/6), and EV-D (1/1). In contrast, the conventional VP4–VP2 RT-PCR detected EV-A in 24/53 specimens (45%), EV-B in 2/6 (33%), and EV-D in 1/1 (100%) specimens in the single-round assay.

At the genotype level within this 60-specimen cohort, the C3R-based RT-PCR detected all CVA6 (24/24), CVA16 (12/12), CVA10 (9/9), and EV-A71 (6/6) specimens, as well as all detected EV-B and EV-D genotypes. In contrast, the OL68-1-based assay showed reduced detection for several EV-A genotypes, including CVA6, CVA10, and EV-A71. These results demonstrate the broad inclusivity of the C3R-based RT-PCR across EV species and genotypes in a single-round format.

The majority of the amplicons produced by the C3R-based RT-PCR were within the expected size range (~750 bp in the first round and ~650 bp in the second round) and were amenable to downstream Sanger sequencing (Methods).

### 3.4. In Silico Coverage and Fitness of C3R (vs. OL68-1)

**Figure 4:**
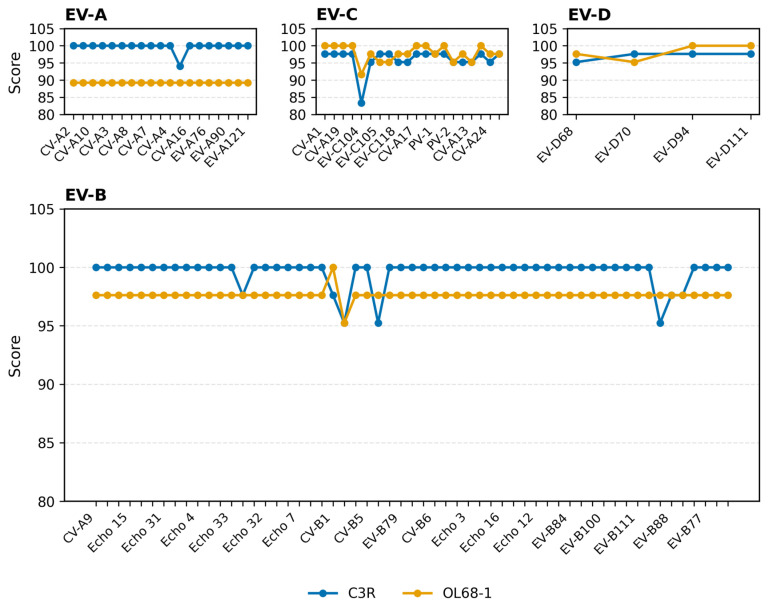
In silico primer–template fitness of C3R and OL68-1 across Enterovirus species A–D. Fitness scores (0–100 scale) for C3R (blue) and OL68-1 (yellow) are presented using a subset of representative genotypes on the x-axis (labels are thinned for readability); trends reflect the full dataset for EV-A, EV-B, EV-C, and EV-D. C3R showed uniformly high fitness in EV-A (predominantly 100) and across most EV-B genotypes, with OL68-1 typically around 97.6 in EV-B (occasionally 88.1). In EV-C and EV-D, both primers generally exhibited high fitness; OL68-1 sometimes achieved perfect matches, whereas C3R typically scored in the mid-to-high 90 s, with EV-C104 being the only notable low C3R outlier (83.33). Comprehensive per-genotype results and alignments are available in Supplementary Table S2. Scores are normalized to a 0–100 scale; values above 100 reflect plotting margins only.

Figure 4 summarizes the in silico primer–template fitness scores of C3R and OL68-1 across representative enterovirus genotypes.

C3R:

Primer–template fitness across 100 EV genotypes showed that 68/100 scored 100 (68.0%) and 31/100 scored 94.1–97.6 (31.0%), indicating perfect or near-perfect matches at the primer 3′ end and predicting robust single-round amplification (Figure 3). The only notable outlier was EV-C104 (EU840733.1), which scored 83.33 due to multiple 3′-proximal mismatches, suggesting reduced amplification efficiency relative to other genotypes (full per-genotype scores are listed in Supplementary Table S2).

OL68-1:

OL68-1 exhibited a species-dependent pattern. In EV-C and EV-D, OL68-1 frequently achieved 97.6–100, occasionally 100, whereas C3R typically fell in the 94–98 range for these species. In EV-B, OL68-1 generally scored around 97.6 (with some at 88.1), but was often lower than C3R, which frequently reached 100 across Echo and CVB representatives; for EV-B81/EV-B87, OL68-1 scored 97.6 or 88.1, while C3R was 95.24. In EV-A, OL68-1 commonly scored ≈ 89.3, consistent with multiple 3′-proximal mismatches and the assay’s reduced empirical sensitivity for EV-A, while C3R was near-uniformly 100. Full per-genotype scores and alignments are provided in Supplementary Table S2.

## 4. Discussion

Major findings

Molecular detection and typing of human enteroviruses have traditionally relied on VP1 sequencing as the definitive standard, based on its strong correlation with serotype and phylogeny [13,25]. However, multiple studies have highlighted practical limitations of VP1-based approaches, including reduced sensitivity in low-viral-load specimens and reliance on nested amplification [13], which limit their suitability for rapid screening and large-scale surveillance [26]. As a result, VP4–VP2-based RT-PCR has long been positioned as a complementary frontline assay that offers operational simplicity, albeit with well-recognized sensitivity limitations, particularly for contemporary Enterovirus A lineages [12,15,27].

Previous evaluations of VP4–VP2 typing have focused mainly on its phylogenetic validity and sequence-based discriminatory power rather than on improving the analytical performance of the amplification step itself [12,15]. Under this framework, whether the sensitivity limitations of conventional VP4–VP2 RT-PCR can be overcome without altering the target region or the assay format remains unresolved. Notably, the OL68-derived VP4 primers introduced by Olive et al. in 1990 [11] represented a landmark contribution developed under severe constraints in available enterovirus sequence data; however, subsequent refinements were necessarily incremental and not informed by the extensive sequence diversity now recognized in contemporary Enterovirus A lineages.

The central contribution of this study is not merely the demonstration of improved detection rates, but the identification of primer–template compatibility at the VP2 3′ region as a critical, previously underappreciated determinant of VP4–VP2 RT-PCR sensitivity. By integrating reference strain experiments, in silico primer fitness evaluation, and clinical specimen testing, we provide a coherent explanation for the long-standing underperformance of conventional VP4–VP2 assays in EV-A–dominant settings and demonstrate that this limitation can be addressed without altering the target region or assay architecture. Consistent with this mechanistic framework, the C3R-based single-round RT-PCR substantially enhanced analytical and diagnostic sensitivity for HFMD-associated enteroviruses, including low-viral-load clinical specimens.

Mechanistic interpretation anchored by reference strain sequences

The superior performance of C3R is mechanistically consistent with its 3′-proximal concordance at the VP2 site, which reduces the penalty incurred at the polymerase-critical end of the primer–template interface. The standard-strain experiments provide an orthogonal anchor for this interpretation: EV-A71 exemplifies the gain under 3′-end mismatch pressure that hampers OL68-1, whereas PV-1 (EV-C) demonstrates that the redesign does not trade sensitivity for breadth in a non-EV-A background. This alignment of the sequence-level rationale and reference-isolate behavior explains why the clinical gains are concentrated in EV-A-rich settings without implying cross-board disadvantages for EV-C and EV-D.

Notably, although C3R carries higher degeneracy than OL68-1 (96 vs. 8 possible sequence variants), its use resulted in less nonspecific amplification in our hands. This indicates that primer–template compatibility at the 3′ critical region exerts a stronger influence on amplification specificity than the nominal degree of degeneracy. Thus, the improved match at the VP2-C3 site appears to offset the theoretical increase in nonspecific binding expected from a more degenerate primer.

The presence of additional non-target amplification products observed with the conventional OL68-1 primer may be attributable, at least in part, to sequence similarity between the primer and regions within the human genome. Sequencing of selected non-target amplicons generated using OL68-1 revealed sequence similarity to entries in publicly available human genome databases, suggesting that incidental priming on human genomic DNA can occur. Notably, this study was not designed to systematically analyze or annotate human genomic sequences, and therefore, further investigation of these regions was beyond its scope.

Additional internal testing under varied annealing conditions did not alter the relative performance of the two primers. Under identical thermocycling conditions, OL68-1 consistently showed reduced amplification relative to C3R, indicating that the observed difference in sensitivity cannot be explained solely by annealing temperature or cycling parameters. These observations further support the conclusion that enhanced primer–template compatibility, particularly at the 3′ end of the primer, is the principal determinant of the improved sensitivity achieved with C3R.

C3R-based RT-PCR: Single-Round Alternative to Conventional VP4–VP2 RT-PCR

The poor single-round performance of conventional VP4–VP2 RT-PCR against EV-A can be mechanistically explained by sequence mismatches at critical sites in contemporary EV-A lineages (e.g., CVA6 clade D and recent EV-A71 strains), as shown by our in silico coverage analysis (Supplementary Table S2) and a concordant external report describing limitations of conventional VP4–VP2-based typing for EV-A compared with alternative typing approaches [27]. Conversely, the C3R primer was rationally redesigned to target the conserved VP2-coding region with minimal mismatches across EV-A, enabling stable amplification even when viral shedding is low (early/late infection and in the CSF) [14] or when RNA is partially degraded. Moreover, while nested VP1 protocols such as CODEHOP PCR can often compensate for sensitivity in low-viral-load specimens, their principal limitations include increased cost, longer turnaround times, added workflow complexity, and a higher risk of carryover contamination compared to single-round assays, which reduces their feasibility for rapid screening or surge-scale surveillance. Notably, the reference-isolate comparison (EV-A71 gain with PV-1 parity) provides direct experimental corroboration of the 3′-end-driven rationale, supporting a C3R-first, single-round strategy without implying a generalized disadvantage in EV-C/EV-D.

Although direct, specimen-by-specimen benchmarking of genotyping concordance between the C3R-based VP4–VP2 assay and VP1-based reference methods such as CODEHOP PCR would be highly informative, the primary aim of the present study was to establish improved analytical and clinical sensitivity relative to the conventional OL68-1-based VP4–VP2 RT-PCR. Systematic evaluation of VP4–VP2 and VP1 typing concordance represents a distinct research objective and will be addressed in a dedicated future study.

Future application to improve the conventional VP4–VP2 manual protocol

Importantly, the insights gained from this redesign process provide a clear roadmap for upgrading existing manual VP4–VP2 RT-PCR workflows. Many laboratories continue to employ OL68-1-based protocols as part of legacy EV typing algorithms. Our findings indicate that simply replacing the reverse primer within these manual protocols—while keeping the remaining steps unchanged—would yield a substantial improvement in sensitivity, particularly for EV-A–dominant HFMD contexts. As several national EV surveillance systems still rely on VP4–VP2 as a front-line assay, we plan to extend our optimization framework to develop a manual version of the C3R-enhanced VP4–VP2 PCR protocol. This will allow institutions with limited access to real-time PCR platforms or automated extraction systems to benefit from the same sensitivity gains demonstrated in the present study, while preserving compatibility with existing sequencing and reporting pipelines.

Practical implications for typing workflows.

Because the improvement is derived from primer–template compatibility rather than cycling heuristics or nested amplification, C3R can be positioned as a first-line, single-round VP4–VP2 RT-PCR that triages specimens to VP1 or whole-capsid sequencing only when identities are borderline or epidemiologically critical. In laboratories where OL68-1 has been retained for historical reasons, the EV-A71/PV-1 pair provides a simple commissioning panel to verify that the expected differential is reproduced locally before a broad rollout. This standard-anchored validation is particularly relevant during HFMD surges, where time-to-result and contamination control favor a single-round strategy.

Importantly, because C3R enables reliable typing using single-round PCR without the need for nested amplification, the workflow becomes both faster and more economical. Eliminating the second PCR reduces reagent use, labor, and hands-on time, while also shortening the overall turnaround by 2–3 h. In practical terms, laboratories can process higher sample volumes at a lower per-specimen cost, which is a critical advantage during seasonal HFMD peaks and public-health surge responses.

Applicability to one-step and real-time formats.

In addition, the C3R primer was also compatible with a one-step RT-PCR workflow. This suggests that C3R is not only advantageous for single-round conventional RT-PCR but is also a promising candidate for real-time PCR assays targeting the VP4–VP2 region. Moving forward, we plan to expand both the number and diversity of clinical specimens (e.g., respiratory samples, sterile-site fluids, and stool) to systematically benchmark C3R performance across one-step and real-time formats and to define its optimal placement within diagnostic algorithms.

Rationale for selecting the VP4–VP2 region and positioning in diagnostic algorithms

Although VP1 sequencing remains the gold standard for definitive typing and genotyping [13,28], supported by a long-standing correlation between the serotype and the VP1 sequence, its relatively large amplicon (~900 bp) and reliance on nested workflows can impair its performance and timeliness in routine clinical specimens.

Our data show that the VP4–VP2 segment, which is shorter and flanked by conserved primer sites, presents a pragmatic target for HFMD-associated EVs. In line with a recent evaluation of VP4–VP2 typing that demonstrated high RT-PCR sensitivity for this region and proposed a 95% amino acid identity threshold for type assignment (with limited exceptions such as PV1–PV2, CVA6/10, and some EV-C types) [15], our single-round PCR using C3R achieved species/genotype coverage across EV-A71, CVA6/10/16, EV-B types, and EV-D68. These VP4 identity data further support that VP4-based typing is robust for EV-A71, CV-A10, and CV-A16, but substantially less reliable for CV-A6, underscoring the importance of cautious interpretation and escalation to VP1-based typing for this genotype.

Coxsackievirus A6 has undergone rapid genetic diversification in recent years [29], with frequent lineage turnover observed in contemporary outbreaks. Under these circumstances, interpretation of VP4–VP2-based typing results can be influenced by the choice of reference sequences used for comparison. We therefore emphasize that the use of appropriately selected, recent CVA6 reference strains—such as KR815992—at the amino acid level can improve the reliability of VP4–VP2-based type assignment, even within this rapidly evolving genotype. While VP4–VP2 remains less robust than VP1 for CVA6 in absolute terms, careful reference selection facilitates more accurate provisional typing and helps inform decisions regarding escalation to VP1-based confirmation.

Beyond analytical sensitivity, the VP4–VP2 junction represents a strategically advantageous target for broad-spectrum enterovirus recognition, owing to the high conservation of primer-binding sites across EV species combined with sufficient downstream variability for molecular typing. By leveraging this conserved junction, the present assay supports inclusive, single-round detection of diverse enterovirus serotypes while maintaining genotype-level resolution. This balance underscores the utility of the VP4–VP2 region as a practical front-line target for translational diagnostic and surveillance applications.

Clinical and public-health significance

In surge settings for HFMD and other EV activities [7,9], a single-round assay that reliably detects EV-A71 and related EVs can shorten reporting by ~2–3 h, support early risk stratification (e.g., neurologic complications), and improve throughput at a lower per-specimen cost. As a Tier-1 screen [15], frontline VP4–VP2 single-round RT-PCR can triage only borderline or epidemiologically critical specimens to VP1 or whole-capsid sequencing, conserving resources for “deep” characterization.

A sensitive front-end screen aligns with international surveillance frameworks that prioritize early detection of severe EV presentations. In Europe, WHO/ENPEN data demonstrate that NPEVs are important causes of paralysis and recommend standardized reporting of severe cases, underscoring the value of a rapid assay before VP1 or whole-capsid sequencing [25]. In the United States, national AFM surveillance and NESS emphasize prompt identification of EV-positive specimens during EV-D68 activity [30], where a fast VP4–VP2 gate helps triage AFM-suspect or ICU cases for deeper genomic investigation. In Japan, notifiable acute encephalitis surveillance and nationwide AFP surveillance (<15 years) reveal large pediatric caseloads with substantial unknown-pathogen proportions, highlighting the importance of rapid EV detection to direct confirmatory sequencing—reinforced by Japan’s nationwide AFM investigation during the 2015 EV-D68 season [31].

Limitations and future directions

Our evaluation focused on the HFMD-related EV-A/EV-B isolates and a small number of EV-D isolates. Performance against less common EV-C/EV-D genotypes warrants broader testing, including in cohorts with CSF-rich meningitis and AFM presentations. Short amplicon typing can be affected by recombination and lineage turnover; thus, borderline identity results (~92–96% AA) should trigger escalation to VP1 or whole-capsid sequencing and be interpreted in conjunction with clinical/epidemiological metadata.

## Figures and Tables

**Table 1:** Primers used in this study.

Primer/Probe Name	Polarity	Sequence (5′ → 3′)	Position (V01149 PV1, Mahoney)	Primer Length (bp)	Reference
EVP2	+	CCTCCGGCCCCTGAATGCGGCTAAT	444–468	25	[18]
EVP4	+	CTACTTTGGGTGTCCGTGTT	541–560	20	[12]
OL68-1	−	GGTAAYTTCCACCACCANCC	1178–1197	20	[11]
C3R	−	TCNGGRAAYTTCCAVYACCA	1181–1200	20	This study
Primer F	+	CCCTGAATGCGGCTAATCC	452–470	19	[19]
Primer R1	−	ATTGTCACCATAAGCAGCCA	577–596	20	[19]
Probe P(FAM)	+	AACCGACTACTTTGGGTGTCCGTGTTTC	535–562	28	[19]
SO224	+	GCIATGYTIGGIACICAYRT	1966–1977	20	[13]
AN89	+	CCAGCACTGACAGCAGYNGARAYNGG	2603–2628	26	[13]
SO222	−	CICCIGGIGGI**AYRWACAT**	2951–2969	19	[13]
AN88	−	TACTGGACCACCTGGNGGN**AYRWACAT**	2951–2969	27	[13]

Ambiguous nucleotide symbols follow standard IUPAC nomenclature. Bold letters indicate consensus-degenerate positions in the primer sequences.

## Data Availability

The data supporting the findings of this study are available within the article and its Supplementary Materials. Additional data underlying this study are derived from routine public health surveillance and contain potentially identifiable information; therefore, these data are not publicly available due to ethical and privacy restrictions. Reasonable requests for access to de-identified data may be considered by the corresponding author, subject to approval by the relevant institutional ethics committees.

## Supplementary Materials

The following supporting information can be downloaded at: https://www.mdpi.com/article/10.3390/v18050527/s1. Table S1: Ct-stratified clinical detection of enteroviruses in the HFMD/herpangina cohort by C3R- and OL68-1-based single-round RT-PCR. Table S2: Primer–template alignments and in silico fitness scores of C3R and OL68-1 across 100 representative enterovirus genotypes. Table S3. VP4 amino acid identity of representative clinical specimens. Figure S1: Representative agarose gel electrophoresis of VP4–VP2 amplicons obtained from clinical specimens with pan-enterovirus real-time RT-PCR Ct values ≥ 35, demonstrating detectable amplification and suitability for downstream Sanger sequencing.

## Abbreviations

The following abbreviations are used in this manuscript:

cDNA	complementary DNA
CODEHOP	consensus-degenerate hybrid oligonucleotide primer
CSF	cerebrospinal fluid
Ct	cycle threshold
CVA	Coxsackievirus A
CVB	Coxsackievirus B
EVs	enteroviruses
FAM	6-carboxyfluorescein
HFMD	hand, foot, and mouth disease
IUPAC	International Union of Pure and Applied Chemistry
LOD	limit of detection
NC	non-coding
PCR	polymerase chain reaction
PV	poliovirus
RT	reverse-transcription
RV	rhinovirus
UTR	untranslated region

## References

[1] Xie Z., Khamrin P., Maneekarn N., Kumthip K. (2024). Epidemiology of Enterovirus Genotypes in Association with Human Diseases. Viruses.

[2] Jartti M., Flodstrom-Tullberg M., Hankaniemi M.M. (2024). Enteroviruses: Epidemic potential, challenges and opportunities with vaccines. J. Biomed. Sci..

[3] ICTV (2026). Family: Picornaviridae Genus: Enterovirus. https://ictv.global/report/chapter/picornaviridae/picornaviridae/enterovirus.

[4] Takahashi S., Metcalf C.J.E., Arima Y., Fujimoto T., Shimizu H., Rogier van Doorn H., Le Van T., Chan Y.F., Farrar J.J., Oishi K. (2018). Epidemic dynamics, interactions and predictability of enteroviruses associated with hand, foot and mouth disease in Japan. J. R. Soc. Interface.

[5] Aswathyraj S., Arunkumar G., Alidjinou E.K., Hober D. (2016). Hand, foot and mouth disease (HFMD): Emerging epidemiology and the need for a vaccine strategy. Med. Microbiol. Immunol..

[6] Yu H., Li X.W., Liu Q.B., Deng H.L., Liu G., Jiang R.M., Deng J.K., Ye Y.Z., Hao J.H., Chen Y.H. (2020). Diagnosis and treatment of herpangina: Chinese expert consensus. World J. Pediatr..

[7] Takechi M., Fukushima W., Nakano T., Inui M., Ohfuji S., Kase T., Ito K., Kondo K., Maeda A., Shimizu H. (2019). Nationwide Survey of Pediatric Inpatients with Hand, Foot, and Mouth Disease, Herpangina, and Associated Complications During an Epidemic Period in Japan: Estimated Number of Hospitalized Patients and Factors Associated with Severe Cases. J. Epidemiol..

[8] Ooi M.H., Wong S.C., Lewthwaite P., Cardosa M.J., Solomon T. (2010). Clinical features, diagnosis, and management of enterovirus 71. Lancet Neurol..

[9] Nagai T., Hanaoka N., Katano H., Konagaya M., Tanaka-Taya K., Shimizu H., Mukai T., Fujimoto T. (2021). A fatal case of acute encephalopathy in a child due to coxsackievirus A2 infection: A case report. BMC Infect. Dis..

[10] Ishii M., Hoshina T., Fujimoto T., Hanaoka N., Konagaya M., Shimbashi R., Takanashi S., Arai S., Tanaka-Taya K., Kusuhara K. (2024). A Pediatric Case of Encephalopathy with Hypoglycemia Induced by Coxsackievirus A4 Infection. Pediatr. Infect. Dis. J..

[11] Olive D.M., Al-Mufti S., Al-Mulla W., Khan M.A., Pasca A., Stanway G., Al-Nakib W. (1990). Detection and differentiation of picornaviruses in clinical samples following genomic amplification. J. Gen. Virol..

[12] Ishiko H., Shimada Y., Yonaha M., Hashimoto O., Hayashi A., Sakae K., Takeda N. (2002). Molecular diagnosis of human enteroviruses by phylogeny-based classification by use of the VP4 sequence. J. Infect. Dis..

[13] Nix W.A., Oberste M.S., Pallansch M.A. (2006). Sensitive, seminested PCR amplification of VP1 sequences for direct identification of all enterovirus serotypes from original clinical specimens. J. Clin. Microbiol..

[14] Fujimoto T., Yoshida S., Munemura T., Taniguchi K., Shinohara M., Nishio O., Chikahira M., Okabe N. (2008). Detection and quantification of enterovirus 71 genome from cerebrospinal fluid of an encephalitis patient by PCR applications. Jpn. J. Infect. Dis..

[15] Kitamura K., Arita M. (2024). Evaluation of VP4-VP2 sequencing for molecular typing of human enteroviruses. PLoS ONE.

[16] National Institute of Infectious Diseases (NIID) (2023). Hand, Foot, and Mouth Disease Pathogen Detection Manual. https://id-info.jihs.go.jp/manuals/pathogen-detection/HFMdis20230704.pdf.

[17] Fujimoto T., Chikahira M., Yoshida S., Ebira H., Hasegawa A., Totsuka A., Nishio O. (2002). Outbreak of central nervous system disease associated with hand, foot, and mouth disease in Japan during the summer of 2000: Detection and molecular epidemiology of enterovirus 71. Microbiol. Immunol..

[18] Rotbart H.A. (1990). Enzymatic RNA amplification of the enteroviruses. J. Clin. Microbiol..

[19] Wolffs P.F., Bruggeman C.A., van Well G.T., van Loo I.H. (2011). Replacing traditional diagnostics of fecal viral pathogens by a comprehensive panel of real-time PCRs. J. Clin. Microbiol..

[20] Ueno M.K., Kitamura K., Nishimura Y., Arita M. (2023). Evaluation of Direct Detection Protocols for Poliovirus from Stool Samples of Acute Flaccid Paralysis Patients. Viruses.

[21] Bustin S.A., Ruijter J.M., van den Hoff M.J.B., Kubista M., Pfaffl M.W., Shipley G.L., Tran N., Rödiger S., Untergasser A., Mueller R. (2025). MIQE 2.0: Revision of the Minimum Information for Publication of Quantitative Real-Time PCR Experiments Guidelines. Clin. Chem..

[22] Johnson A.D. (2010). An extended IUPAC nomenclature code for polymorphic nucleic acids. Bioinformatics.

[23] Stadhouders R., Pas S.D., Anber J., Voermans J., Mes T.H., Schutten M. (2010). The effect of primer-template mismatches on the detection and quantification of nucleic acids using the 5’ nuclease assay. J. Mol. Diagn..

[24] Rejali N.A., Moric E., Wittwer C.T. (2018). The Effect of Single Mismatches on Primer Extension. Clin. Chem..

[25] Oberste M.S., Maher K., Kilpatrick D.R., Pallansch M.A. (1999). Molecular evolution of the human enteroviruses: Correlation of serotype with VP1 sequence and application to picornavirus classification. J. Virol..

[26] Majumdar M., Celma C., Pegg E., Polra K., Dunning J., Martin J. (2021). Detection and Typing of Human Enteroviruses from Clinical Samples by Entire-Capsid Next Generation Sequencing. Viruses.

[27] Cardosa M.J., Perera D., Brown B.A., Cheon D., Chan H.M., Chan K.P., Cho H., McMinn P. (2003). Molecular epidemiology of human enterovirus 71 strains and recent outbreaks in the Asia-Pacific region: Comparative analysis of the VP1 and VP4 genes. Emerg. Infect. Dis..

[28] Thoelen I., Moës E., Lemey P., Mostmans S., Wollants E., Lindberg A.M., Vandamme A.M., Van Ranst M. (2004). Analysis of the serotype and genotype correlation of VP1 and the 5’ noncoding region in an epidemiological survey of the human enterovirus B species. J. Clin. Microbiol..

[29] Song Y., Zhang Y., Han Z., Xu W., Xiao J., Wang X., Wang J., Yang J., Yu Q., Yu D. (2020). Genetic recombination in fast-spreading coxsackievirus A6 variants: A potential role in evolution and pathogenicity. Virus Evol..

[30] Whitehouse E.R., Lopez A., English R., Getachew H., Ng T.F.F., Emery B., Rogers S., Kidd S. (2024). Surveillance for Acute Flaccid Myelitis—United States, 2018–2022. MMWR Morb. Mortal. Wkly. Rep..

[31] Chong P.F., Kira R., Mori H., Okumura A., Torisu H., Yasumoto S., Shimizu H., Fujimoto T., Hanaoka N., Kusunoki S. (2018). Clinical Features of Acute Flaccid Myelitis Temporally Associated with an Enterovirus D68 Outbreak: Results of a Nationwide Survey of Acute Flaccid Paralysis in Japan, August–December 2015. Clin. Infect. Dis..

