# Supplementary material for: Enterovirus Testing in Hand, Foot, and Mouth Disease and Herpangina: A Highly Sensitive Single-Round VP4–VP2 Reverse-Transcription Polymerase Chain Reaction Assay with a Redesigned Reverse Primer
Source: Viruses. 2026 Apr 30;18(5):527. doi: 10.3390/v18050527 (PMC13211326; doi:10.3390/v18050527)
Supplement: Supplementary file 1 [file viruses-18-00527-s001.zip › Supplemental Figure S1.pdf]

Type	Ct	VP4–VP2 single-round PCR (C3R)	Sample ID	Electrophoresis				Sanger sequence & BLAST		
				single PCR	Results	nested PCR	Results	Length	Top Hit	Identity
CVA6	35.08	+	14599	Lane 11	Band detected	Not done	not done	708bp	AB678778.1	99.15%
CVA6	35.28	+	17138	Lane 32	Band detected	Lane 67	Band detected	616bp	MT814458.1	97.89%
CVA6	35.66	+	14432	Lane 3	Band detected	Not done	not done	708bp	LC126157.1	100%
CVA16	37.74	+	16933	Lane 30	Band detected	Lane 66	Band detected	615bp	LC506458.1	99.51%
CVA10	37.78	+	19829	Lane 50	Band detected	Lane 78	Band detected	615bp	PP078992.1	98.05%
EV-A71	45	+	15125	Lane 13	Band detected	Lane 58	Band detected	618bp	LC626875.1	99.51%
CVA6	45	-	20093	Lane 54	Band not detected	Lane 81	Band detected	616bp	LC789938.1	100%
CVB5	38.41	+	11852	Lane 90	Band detected	Lane 100	Band detected	Not done	Not done	Not done

Single-round VP4–VP2 RT-PCR (C3R) : (A) Single-round PCR

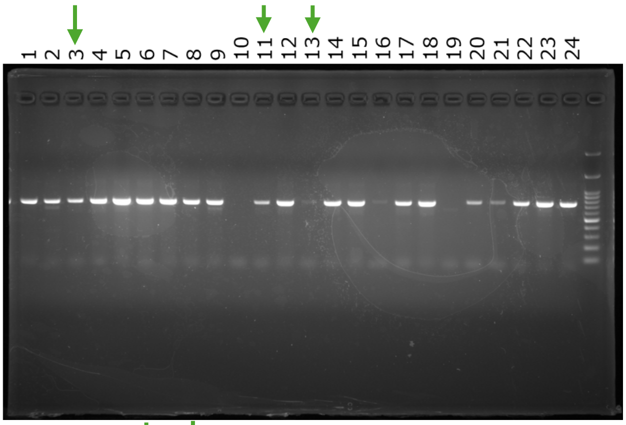

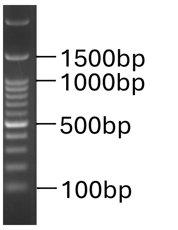

Lane 3: CVA6, Ct 35.66
Lane11: CVA6, Ct 35.08
Lane 13: EV-A71, Ct 45

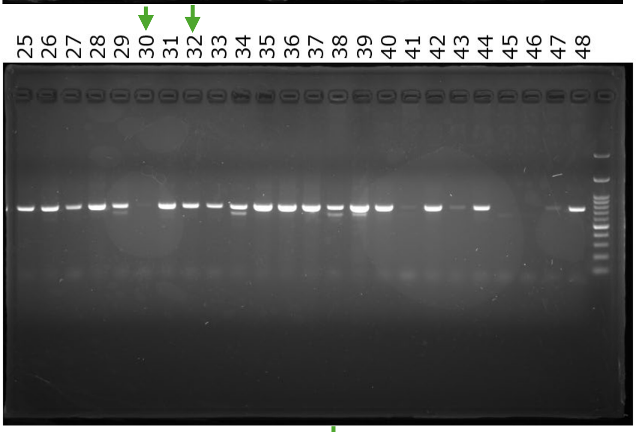

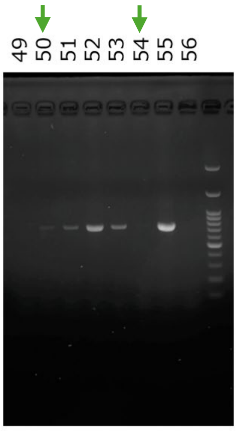

Lane 30: CVA16, Ct 37.74
Lane 32: CVA6, Ct 35.28
Lane 50: CVA10, Ct 37.78
Lane 54: CVA6, Ct 45

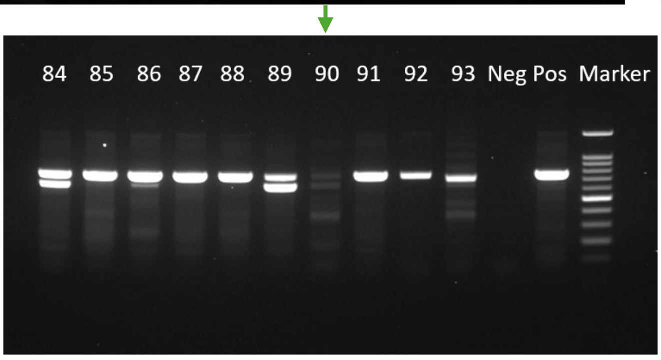

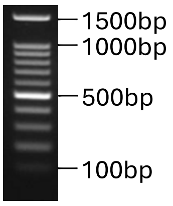

Lane 90: CVB5, Ct 38.41

Semi-nested VP4–VP2 RT-PCR (C3R): (B) Semi-nested PCR

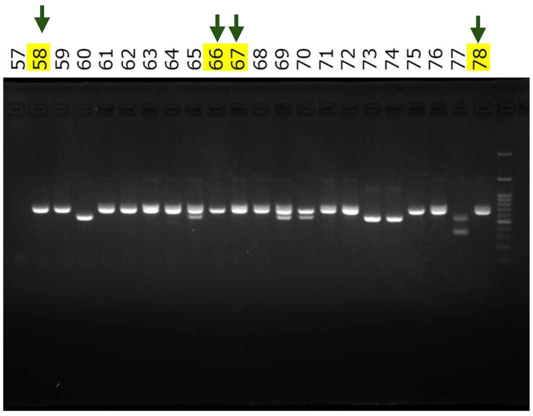

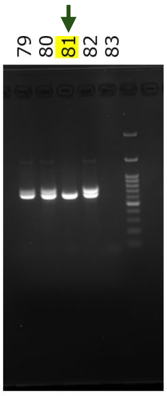

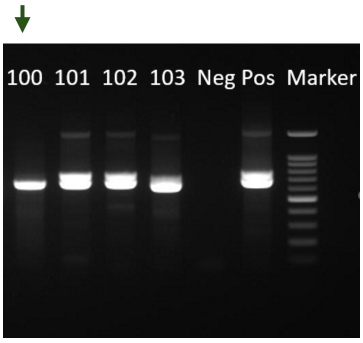

Lane 58: EV-A71, Ct 45
Lane 66: CVA16, Ct 37.74
Lane 67: CVA6, Ct 35.28
Lane 78: CVA10, Ct 37.78

Lane 81: CVA6, Ct 45

Lane 100: CVB5 Ct 38.41

Supplementary Figure S1.

Representative agarose gel electrophoresis of VP4–VP2 amplicons obtained from clinical specimens with pan-enterovirus real-time RT-PCR Ct values ≥35. Red labels indicate results obtained with the C3R-based workflow.

Amplicons were generated using the C3R-based single-round VP4–VP2 RT-PCR and, where indicated, further amplified by semi-nested PCR. Selected representative amplicons that yielded detectable bands at the expected size were subjected to Sanger sequencing, and corresponding typing results are summarized in the accompanying table.

Molecular size markers used were 100bp DNA Ladder H3 RTU (GeneDirex Inc) or ExcelBand 100 bp DNA Ladder (DM2100; SMO BIO), depending on gel batch availability.

“Not done” indicates that sequencing was not attempted due to insufficient amplicon yield or prioritization of representative samples.
