# Supplementary material for: Enterovirus Testing in Hand, Foot, and Mouth Disease and Herpangina: A Highly Sensitive Single-Round VP4–VP2 Reverse-Transcription Polymerase Chain Reaction Assay with a Redesigned Reverse Primer
Source: Viruses. 2026 Apr 30;18(5):527. doi: 10.3390/v18050527 (PMC13211326; doi:10.3390/v18050527)
Supplement: Supplementary file 1 [file viruses-18-00527-s001.zip › Supplementary Table S1.pdf]

Supplementary Table S1. Ct-stratified clinical detection of enteroviruses in the HFMD/herpangina cohort by C3R- and OL68-1-based single-round RT-PCR.

Type	C3R–single round PCR	OL68–1–single round PCR extra band	Species	Ct value	Year of specimen collection	Specimen	Ct Range	Primer	Positive rate	95% CI(Clopper–Pearson)
CV–A16	+	+	A	22.2	2019	Oropharyngeal swab	**<25**	C3R	100% (9/9)	66.4–100.0%
CV–A6	+	+	A	22.2	2015	Oropharyngeal swab		OL68–1	88.9% (8/9)	51.8–99.7%
CV–A6	+	+	A	22.3	2017	Nasopharyngeal swab				
CV–A10	+	+	A	22.8	2017	Oropharyngeal swab				
CV–A10	+	+	A	23.3	2017	Oropharyngeal swab				
CV–A6	+	–	A	23.7	2019	Oropharyngeal swab				
CV–A9	+	+	B	24.1	2016	Oropharyngeal swab				
CV–A10	+	+	A	24.2	2016	Oropharyngeal swab				
CV–A5	+	+	A	24.8	2019	Oropharyngeal swab				
CV–A16	+	+	A	25.2	2019	Oropharyngeal swab	**25–29.9**	C3R	100% (17/17)	80.5–100.0%
EV–A71	+	+	A	26.2	2018	Oropharyngeal swab		OL68–1	52.9% (9/17)	27.8–77.0%
CV–A6	+	–	A	26.3	2015	Oropharyngeal swab				
CV–A6	+	+	A	26.6	2011	Oropharyngeal swab				
CV–A16	+	+	A	26.7	2019	Saliva				
EV–A71	+	+	A	27.0	2017	Oropharyngeal swab				
EV–A71	+	–	A	27.6	2018	Oropharyngeal swab				
CV–A6	+	+	A	27.9	2019	Stool				
CV–A10	+	–	A	27.9	2014	Oropharyngeal swab				
CV–B3	+	–	B	28.3	2014	Oropharyngeal swab				
CV–A16	+	–	A	28.4	2011	Oropharyngeal swab				
Echo18	+	–	B	28.4	2019	Stool				
CV–A6	+	+	A	28.6	2022	Saliva				
CV–A4	+	+	A	28.8	2016	Oropharyngeal swab				
CV–A16	+	+	A	29.0	2019	Saliva				
CV–A16	+	–	A	29.2	2014	Oropharyngeal swab				
Echo 9	+	–	B	29.7	2016	Stool				
CV–A6	+	+	A	30.1	2012	Oropharyngeal swab	**30–34.9**	C3R	100% (26/26)	86.8–100.0%
CV–A16	+	–	A	30.2	2019	Saliva		OL68–1	34.6% (9/26)	17.2–55.7%
CV–A10	+	+	A	30.4	2011	Oropharyngeal swab				
EVD68	+	+	D	30.5	2018	Nasal secretion				
CV–A6	+	–	A	30.7	2011	Oropharyngeal swab				
CV–A6	+	–	A	31.0	2019	Oropharyngeal swab				
CV–A10	+	–	A	31.0	2011	Oropharyngeal swab				
CV–A6	+	+	A	31.0	2013	Oropharyngeal swab				
CV–A6	+	+	A	31.2	2013	Oropharyngeal swab				
CV–A6	+	–	A	31.2	2013	Oropharyngeal swab				
CV–A6	+	–	A	31.2	2012	Oropharyngeal swab				
CV–A6	+	–	A	31.4	2012	Oropharyngeal swab				
CV–A6	+	–	A	31.7	2011	Oropharyngeal swab				
CV–A10	+	–	A	31.8	2011	Oropharyngeal swab				
CV–A16	+	+	A	31.9	2013	Oropharyngeal swab				
CV–A6	+	+	A	32.0	2015	Oropharyngeal swab				
CV–A16	+	+	A	32.0	2013	Oropharyngeal swab				
EV–A71	+	–	A	32.4	2013	Oropharyngeal swab				
CV–A10	+	+	A	33.8	2020	Oropharyngeal swab				
EV–A71	+	–	A	34.0	2013	Oropharyngeal swab				
CV–A16	+	–	A	34.0	2019	Saliva				
CV–A6	+	–	A	34.2	2015	Oropharyngeal swab				
CV–A6	+	–	A	34.3	2022	Saliva				
CV–A16	+	–	A	34.3	2019	Saliva				
CV–A6	+	–	A	34.4	2013	Oropharyngeal swab				
Echo30	+	–	B	34.7	2019	Cerebrospinal fluid				
CV–A6	+	–	A	35.1	2011	Oropharyngeal swab	**35–39.9**	C3R	100% (6/6)	54.1–100.0%
CV–A6	+	–	A	35.3	2015	Oropharyngeal swab		OL68–1	16.7%(1/6)	0.4–64.1%
CV–A6	+	–	A	35.7	2011	Oropharyngeal swab				
CV–A16	+	–	A	37.7	2015	Oropharyngeal swab				
CV–A10	+	–	A	37.8	2020	Oropharyngeal swab				
CVB5	+	+	B	38.4	2016	Nasal secretion				
EV–A71	+	–	A	45.0	2012	Oropharyngeal swab	**45**	C3R	50% (1/2)	1.3–98.7%
CV–A6	–	–	A	45.0	2022	Saliva		OL68–1	0% (0/2)	0.0–84.2%

Detection rates are shown with exact 95% confidence intervals (Clopper–Pearson method).
