# Supplementary material for: Enterovirus Testing in Hand, Foot, and Mouth Disease and Herpangina: A Highly Sensitive Single-Round VP4–VP2 Reverse-Transcription Polymerase Chain Reaction Assay with a Redesigned Reverse Primer
Source: Viruses. 2026 Apr 30;18(5):527. doi: 10.3390/v18050527 (PMC13211326; doi:10.3390/v18050527)
Supplement: Supplementary file 1 [file viruses-18-00527-s001.zip › Supplementary Table S2.pdf]

Supplementary Table S2: Primer–template alignments and in silico fitness scores of C3R and OL68-1 across 100 representative Enterovirus genotypes.

					C3R	TCNGGRAAYTTCCAVYACCA																					OL68-1	GGTAAYTTCCACCACCANCC																			
						1	2	3	4	5	6	7	8	9	10	11	12	13	14	15	16	17	18	19	20			1	2	3	4	5	6	7	8	9	10	11	12	13	14	15	16	17	18	19	20
Species	No	Accession	Virus	Sequence	Score	T	G	G	T	R	B	T	G	G	A	A	R	T	T	Y	C	C	N	G	A	Sequence	Score	G	G	N	T	G	G	T	G	G	T	G	G	A	A	R	T	T	A	C	C
EV-A	1	NC_038306.1	CV-A2	tggtattggaagttccctga	100	t	g	g	t	a	t	t	g	g	a	a	g	t	t	c	c	c	t	g	a	ggttggtattggaagttccc	89.29	g	g	t	t	g	g	t	a	t	t	g	g	a	a	g	t	t	c	c	c
EV-A	2	AY421764.1	CV-A6	tggtattggaaattccctga	100	t	g	g	t	a	t	t	g	g	a	a	a	t	t	c	c	c	t	g	a	ggctggtactggaaattccc	89.29	g	g	c	t	g	g	t	a	c	t	g	g	a	a	a	t	t	c	c	c
EV-A	3	AY421767.1	CV-A10	tggtattggaagtttccaga	100	t	g	g	t	a	t	t	g	g	a	a	g	t	t	t	c	c	a	g	a	ggatggtattggaagttccc	89.29	g	g	a	t	g	g	t	a	t	t	g	g	a	a	g	t	t	c	c	c
EV-A	4	AY421768.1	CV-A12	tggtattggaaattcccaga	100	t	g	g	t	a	t	t	g	g	a	a	a	t	t	c	c	c	a	g	a	ggttggtattggaaattccc	89.29	g	g	t	t	g	g	t	a	t	t	g	g	a	a	a	t	t	c	c	c
EV-A	5	AY421761.1	CV-A3	tggtactggaaattccctga	100	t	g	g	t	a	c	t	g	g	a	a	a	t	t	c	c	c	t	g	a	gggtggtattggaaattccc	89.29	g	g	g	t	g	g	t	a	t	t	g	g	a	a	a	t	t	c	c	c
EV-A	6	U22521.1	EV-A71	tggtactggaagtttcccga	100	t	g	g	t	a	c	t	g	g	a	a	g	t	t	t	c	c	c	g	a	gggtggtactggaagttccc	89.29	g	g	g	t	g	g	t	a	c	t	g	g	a	a	g	t	t	c	c	c
EV-A	7	AY421766.1	CV-A8	tggtattggaagtttcctga	100	t	g	g	t	a	t	t	g	g	a	a	g	t	t	t	c	c	t	g	a	ggatggtattggaagtttcc	89.29	g	g	a	t	g	g	t	a	t	t	g	g	a	a	g	t	t	t	c	c
EV-A	8	AY421763.1	CV-A5	tggtattggaaattccctga	100	t	g	g	t	a	t	t	g	g	a	a	a	t	t	c	c	c	t	g	a	gggtggtattggaagtttcc	89.29	g	g	g	t	g	g	t	a	t	t	g	g	a	a	g	t	t	t	c	c
EV-A	9	AY421765.1	CV-A7	tggtactggaagttcccaga	100	t	g	g	t	a	c	t	g	g	a	a	g	t	t	c	c	c	a	g	a	gggtggtattggaaattccc	89.29	g	g	g	t	g	g	t	a	t	t	g	g	a	a	a	t	t	c	c	c
EV-A	10	LK021688	EV-A120	tggtactggaaattcccaga	100	t	g	g	t	a	c	t	g	g	a	a	a	t	t	c	c	c	a	g	a	ggctggttttggaaattccc	89.29	g	g	c	t	g	g	t	t	t	t	g	g	a	a	a	t	t	c	c	c
EV-A	11	AY421762.1	CV-A4	tggtattggaagttcccaga	100	t	g	g	t	a	t	t	g	g	a	a	g	t	t	c	c	c	a	g	a	ggttggtattggaaattccc	89.29	g	g	t	t	g	g	t	a	t	t	g	g	a	a	a	t	t	c	c	c
EV-A	12	AY421769.1	CV-A14	tggttttggaaattcccgga	94.05	t	g	g	t	t	t	t	g	g	a	a	a	t	t	c	c	c	g	g	a	gggtggtactggaagtttcc	89.29	g	g	g	t	g	g	t	a	c	t	g	g	a	a	g	t	t	t	c	c
EV-A	13	U05876.1	CV-A16	tggtattggaaattccccga	100	t	g	g	t	a	t	t	g	g	a	a	a	t	t	c	c	c	c	g	a	ggatggtactggaaattccc	89.29	g	g	a	t	g	g	t	a	c	t	g	g	a	a	a	t	t	c	c	c
EV-A	14	KU355876	EV-A114	tggtattggaaattcccaga	100	t	g	g	t	a	t	t	g	g	a	a	a	t	t	c	c	c	a	g	a	ggatggtactggaagtttcc	89.29	g	g	a	t	g	g	t	a	c	t	g	g	a	a	g	t	t	t	c	c
EV-A	15	AY697458	EV-A76	tggtactggaaattcccgga	100	t	g	g	t	a	c	t	g	g	a	a	a	t	t	c	c	c	g	g	a	ggatggtactggaaattccc	89.29	g	g	a	t	g	g	t	a	c	t	g	g	a	a	a	t	t	c	c	c
EV-A	16	AY697459	EV-A89	tggtactggaagtttcctga	100	t	g	g	t	a	c	t	g	g	a	a	g	t	t	t	c	c	t	g	a	ggttggtattggaaattccc	89.29	g	g	t	t	g	g	t	a	t	t	g	g	a	a	a	t	t	c	c	c
EV-A	17	AY697460	EV-A90	tggtactggaaattccctga	100	t	g	g	t	a	c	t	g	g	a	a	a	t	t	c	c	c	t	g	a	gggtggtattggaaattccc	89.29	g	g	g	t	g	g	t	a	t	t	g	g	a	a	a	t	t	c	c	c
EV-A	18	AY697461	EV-A91	tggtattggaaattccctga	100	t	g	g	t	a	t	t	g	g	a	a	a	t	t	c	c	c	t	g	a	gggtggtactggaaattccc	89.29	g	g	g	t	g	g	t	a	c	t	g	g	a	a	a	t	t	c	c	c
EV-A	19	KU355877	EV-A121	tggtattggaagtttccaga	100	t	g	g	t	a	t	t	g	g	a	a	g	t	t	t	c	c	a	g	a	gggtggtattggaagtttcc	89.29	g	g	g	t	g	g	t	a	t	t	g	g	a	a	g	t	t	t	c	c
EV-B	1	D00627.1	CV-A9	tggtggtggaagttcccaga	100	t	g	g	t	g	g	t	g	g	a	a	g	t	t	c	c	c	a	g	a	gggtggtggtggaagttccc	97.62	g	g	g	t	g	g	t	g	g	t	g	g	a	a	g	t	t	c	c	c
EV-B	2	AY302547.1	Echo 21	tggtggtggaagttccctga	100	t	g	g	t	g	g	t	g	g	a	a	g	t	t	c	c	c	t	g	a	ggctggtggtggaagttccc	97.62	g	g	c	t	g	g	t	g	g	t	g	g	a	a	g	t	t	c	c	c
EV-B	3	AY302545.1	Echo 2	tggtggtggaaatttcccga	100	t	g	g	t	g	g	t	g	g	a	a	a	t	t	t	c	c	c	g	a	gggtggtggtggaaatttcc	97.62	g	g	g	t	g	g	t	g	g	t	g	g	a	a	a	t	t	t	c	c
EV-B	4	AY302541.1	Echo 15	tggtggtggaagttccctga	100	t	g	g	t	g	g	t	g	g	a	a	g	t	t	c	c	c	t	g	a	gggtggtggtggaagttccc	97.62	g	g	g	t	g	g	t	g	g	t	g	g	a	a	g	t	t	c	c	c
EV-B	5	AY843298.1	EV-B80	tggtggtggaaatttccaga	100	t	g	g	t	g	g	t	g	g	a	a	a	t	t	t	c	c	a	g	a	ggatggtggtggaaatttcc	97.62	g	g	a	t	g	g	t	g	g	t	g	g	a	a	a	t	t	t	c	c
EV-B	6	AF083069.1	Echo 5	tggtggtggaagtttccaga	100	t	g	g	t	g	g	t	g	g	a	a	g	t	t	t	c	c	a	g	a	gggtggtggtggaagtttcc	97.62	g	g	g	t	g	g	t	g	g	t	g	g	a	a	g	t	t	t	c	c
EV-B	7	AY302554.1	Echo 31	tggtggtggaaatttcctga	100	t	g	g	t	g	g	t	g	g	a	a	a	t	t	t	c	c	t	g	a	ggatggtggtggaaatttcc	97.62	g	g	a	t	g	g	t	g	g	t	g	g	a	a	a	t	t	t	c	c
EV-B	8	X84981.1	Echo 9	tggtggtggaagtttcctga	100	t	g	g	t	g	g	t	g	g	a	a	g	t	t	t	c	c	t	g	a	ggttggtggtggaagtttcc	97.62	g	g	t	t	g	g	t	g	g	t	g	g	a	a	g	t	t	t	c	c
EV-B	9	AF317694.1	Echo 18	tggtggtggaagttccctga	100	t	g	g	t	g	g	t	g	g	a	a	g	t	t	c	c	c	t	g	a	gggtggtggtggaagttccc	97.62	g	g	g	t	g	g	t	g	g	t	g	g	a	a	g	t	t	c	c	c
EV-B	10	AY302557.1	Echo 4	tggtggtggaaattccccga	100	t	g	g	t	g	g	t	g	g	a	a	a	t	t	c	c	c	c	g	a	ggatggtggtggaaattccc	97.62	g	g	a	t	g	g	t	g	g	t	g	g	a	a	a	t	t	c	c	c
EV-B	11	AY302544.1	Echo 19	tggtggtggaagtttccaga	100	t	g	g	t	g	g	t	g	g	a	a	g	t	t	t	c	c	a	g	a	ggatggtggtggaagtttcc	97.62	g	g	a	t	g	g	t	g	g	t	g	g	a	a	g	t	t	t	c	c
EV-B	12	AY302546.1	Echo 20	tggtggtggaaatttcccga	100	t	g	g	t	g	g	t	g	g	a	a	a	t	t	t	c	c	c	g	a	gggtggtggtggaaatttcc	97.62	g	g	g	t	g	g	t	g	g	t	g	g	a	a	a	t	t	t	c	c
EV-B	13	AY302556.1	Echo 33	tggtggtggaaattcccaga	100	t	g	g	t	g	g	t	g	g	a	a	a	t	t	c	c	c	a	g	a	gggtggtggtggaaattccc	97.62	g	g	g	t	g	g	t	g	g	t	g	g	a	a	a	t	t	c	c	c
EV-B	14	AY302548.1	Echo 24	tggtggtggaagtttcccaa	97.62	t	g	g	t	g	g	t	g	g	a	a	g	t	t	t	c	c	c	a	a	ggttggtggtggaagtttcc	97.62	g	g	t	t	g	g	t	g	g	t	g	g	a	a	g	t	t	t	c	c
EV-B	15	AY302560.1	EV-69	tggtggtggaagttcccaga	100	t	g	g	t	g	g	t	g	g	a	a	g	t	t	c	c	c	a	g	a	ggatggtggtggaagttccc	97.62	g	g	a	t	g	g	t	g	g	t	g	g	a	a	g	t	t	c	c	c
EV-B	16	AY302555.1	Echo 32	tggtggtggaagttcccaga	100	t	g	g	t	g	g	t	g	g	a	a	g	t	t	c	c	c	a	g	a	ggctggtggtggaagttccc	97.62	g	g	c	t	g	g	t	g	g	t	g	g	a	a	g	t	t	c	c	c
EV-B	17	AY302552.1	Echo 29	tggtggtggaaatttcctga	100	t	g	g	t	g	g	t	g	g	a	a	a	t	t	t	c	c	t	g	a	gggtggtggtggaaatttcc	97.62	g	g	g	t	g	g	t	g	g	t	g	g	a	a	a	t	t	t	c	c
EV-B	18	AF241359.1	EV-B73	tggtggtggaagttcccaga	100	t	g	g	t	g	g	t	g	g	a	a	g	t	t	c	c	c	a	g	a	gggtggtggtggaagttccc	97.62	g	g	g	t	g	g	t	g	g	t	g	g	a	a	g	t	t	c	c	c
EV-B	19	AY302559.1	Echo 7	tggtggtggaaattcccaga	100	t	g	g	t	g	g	t	g	g	a	a	a	t	t	c	c	c	a	g	a	gggtggtggtggaaattccc	97.62	g	g	g	t	g	g	t	g	g	t	g	g	a	a	a	t	t	c	c	c
EV-B	20	AY843301.1	EV-B83	tggtggtggaagtttccgga	100	t	g	g	t	g	g	t	g	g	a	a	g	t	t	t	c	c	g	g	a	gggtggtggtggaagtttcc	97.62	g	g	g	t	g	g	t	g	g	t	g	g	a	a	g	t	t	t	c	c
EV-B	21	AY302543.1	Echo 17	tggtggtggaaatttccaga	100	t	g	g	t	g	g	t	g	g	a	a	a	t	t	t	c	c	a	g	a	ggatggtggtggaaatttcc	97.62	g	g	a	t	g	g	t	g	g	t	g	g	a	a	a	t	t	t	c	c
EV-B	22	M16560.1	CV-B1	tggtggtggaagttaccaga	97.62	t	g	g	t	g	g	t	g	g	a	a	g	t	t	a	c	c	a	g	a	ggttggtggtggaagttacc	100	g	g	t	t	g	g	t	g	g	t	g	g	a	a	g	t	t	a	c	c
EV-B	23	M16572.1	CV-B3	tggtggtggaagctgcccga	95.24	t	g	g	t	g	g	t	g	g	a	a	g	c	t	g	c	c	c	g	a	ggatggtggtggaagctgcc	95.24	g	g	a	t	g	g	t	g	g	t	g	g	a	a	g	c	t	g	c	c
EV-B	24	X80059.1	Echo 11	tggtggtggaaattcccgga	100	t	g	g	t	g	g	t	g	g	a	a	a	t	t	c	c	c	g	g	a	gggtggtggtggaaattccc	97.62	g	g	g	t	g	g	t	g	g	t	g	g	a	a	a	t	t	c	c	c
EV-B	25	AF114383.1	CV-B5	tggtggtggaagtttcccga	100	t	g	g	t	g	g	t	g	g	a	a	g	t	t	t	c	c	c	g	a	ggatggtggtggaagtttcc	97.62	g	g	a	t	g	g	t	g	g	t	g	g	a	a	g	t	t	t	c	c
EV-B	26	AY843299.1	EV-B81	tggtggtggaaatttcctat	95.24	t	g	g	t	g	g	t	g	g	a	a	a	t	t	t	c	c	t	a	t	ggctggtggtggaaatttcc	97.62	g	g	c	t	g	g	t	g	g	t	g	g	a	a	a	t	t	t	c	c
EV-B	27	AF085363.1	CV-B2	tggtggtggaagtttcccga	100	t	g	g	t	g	g	t	g	g	a	a	g	t	t	t	c	c	c	g	a	ggttggtggtggaagtttcc	97.62	g	g	t	t	g	g	t	g	g	t	g	g	a	a	g	t	t	t	c	c
EV-B	28	AY843297.1	EV-B79	tggtggtggaaattccctga	100	t	g	g	t	g	g	t	g	g	a	a	a	t	t	c	c	c	t	g	a	ggttggtggtggaaattccc	97.62	g	g	t	t	g	g	t	g	g	t	g	g	a	a	a	t	t	c	c	c
EV-B	29	AY843300.1	EV-B82	tggtggtggaagtttcccga	100	t	g	g	t	g	g	t	g	g	a	a	g	t	t	t	c	c	c	g	a	ggttggtggtggaagtttcc	97.62	g	g	t	t	g	g	t	g	g	t	g	g	a	a	g	t	t	t	c	c
EV-B	30	X05690.1	CV-B4	tggtggtggaagttcccaga	100	t	g	g	t	g	g	t	g	g	a	a	g	t	t	c	c	c	a	g	a	gggtggtggtggaagttccc	97.62	g	g	g	t	g	g	t	g	g	t	g	g	a	a	g	t	t	c	c	c
EV-B	31	AF105342.1	CV-B6	tggtggtggaagtttcctga	100	t	g	g	t	g	g	t	g	g	a	a	g	t	t	t	c	c	t	g	a	gggtggtggtggaagtttcc	97.62	g	g	g	t	g	g	t	g	g	t	g	g	a	a	g	t	t	t	c	c
EV-B	32	AY302549.1	Echo 25	tggtggtggaaattcccaga	100	t	g	g	t	g	g	t	g	g	a	a	a	t	t	c	c	c	a	g	a	ggatggtggtggaaattccc	97.62	g	g	a	t	g	g	t	g	g	t	g	g	a	a	a	t	t	c	c	c
EV-B	33	AY302539.1	Echo 13	tggtggtggaagttcccaga	100	t	g	g	t	g	g	t	g	g	a	a	g	t	t	c	c	c	a	g	a	gggtggtggtggaagttccc	97.62	g	g	g	t	g	g	t	g	g	t	g	g	a	a	g	t	t	c	c	c
EV-B	34	AY302553.1	Echo 3	tggtggtggaagttccccga	100	t	g	g	t	g	g	t	g	g	a	a	g	t	t	c	c	c	c	g	a	ggatggtggtggaagttccc	97.62	g	g	a	t	g	g	t	g	g	t	g	g	a	a	g	t	t	c	c	c
EV-B	35	AY302558.1	Echo 6	tggtggtggaagttccctga	100	t	g	g	t	g	g	t	g	g	a	a	g	t	t	c	c	c	t	g	a	gggtggtggtggaagttccc	97.62	g	g	g	t	g	g	t	g	g	t	g	g	a	a	g	t	t	c	c	c
EV-B	36	AY302540.1	Echo 14	tggtggtggaaattccccga	100	t	g	g	t	g	g	t	g	g	a	a	a	t	t	c	c	c	c	g	a	ggatggtggtggaaattccc	97.62	g	g	a	t	g	g	t	g	g	t	g	g	a	a	a	t	t	c	c	c
EV-B	37	AY302542.1	Echo 16	tggtggtggaagtttccaga	100	t	g	g	t	g	g	t	g	g	a	a	g	t	t	t	c	c	a	g	a	ggatggtggtggaagtttcc	97.62	g	g	a	t	g	g	t	g	g	t	g	g	a	a	g	t	t	t	c	c
EV-B	38	AY302550.1	Echo 26	tggtggtggaagttcccaga	100	t	g	g	t	g	g	t	g	g	a	a	g	t	t	c	c	c	a	g	a	ggatggtggtggaagttccc	97.62	g	g	a	t	g	g	t	g	g	t	g	g	a	a	g	t	t	c	c	c
EV-B	39	AY302551.1	Echo 27	tggtggtggaagttccccga	100	t	g	g	t	g	g	t	g	g	a	a	g	t	t	c	c	c	c	g	a	gggtggtggtggaagttccc	97.62	g	g	g	t	g	g	t	g	g	t	g	g	a	a	g	t	t	c	c	c
EV-B	40	X79047.1	Echo 12	tggtggtggaagtttccgga	100	t	g	g	t	g	g	t	g	g	a	a	g	t	t	t	c	c	g	g	a	gggtggtggtggaagtttcc	97.62	g	g	g	t	g	g	t	g	g	t	g	g	a	a	g	t	t	t	c	c
EV-B	41	AF029859.2	Echo 1	tggtggtggaagtttcccga	100	t	g	g	t	g	g	t	g	g	a	a	g	t	t	t	c	c	c	g	a	ggttggtggtggaagtttcc	97.62	g	g	t	t	g	g	t	g	g	t	g	g	a	a	g	t	t	t	c	c
EV-B	42	AY843308.1	EV-B101	tggtggtggaagtttccaga	100	t	g	g	t	g	g	t	g	g	a	a	g	t	t	t	c	c	a	g	a	gggtggtggtggaagtttcc	97.62	g	g	g	t	g	g	t	g	g	t	g	g	a	a	g	t	t	t	c	c
EV-B	43	DQ902712.1	EV-B84	tggtggtggaagttccctga	100	t	g	g	t	g	g	t	g	g	a	a	g	t	t	c	c	c	t	g	a	ggttggtggtggaagttccc	97.62	g	g	t	t	g	g	t	g	g	t	g	g	a	a	g	t	t	c	c	c
EV-B	44	AF162711.1	Echo 30	tggtggtggaagttcccaga	100	t	g	g	t	g	g	t	g	g	a	a	g	t	t	c	c	c	a	g	a	gggtggtggtggaagttccc	97.62	g	g	g	t	g	g	t	g	g	t	g	g	a	a	g	t	t	c	c	c
EV-B	45	AY556057.1	EV-B74	tggtggtggaagtttccaga	100	t	g	g	t	g	g	t	g	g	a	a	g	t	t	t	c	c	a	g	a	gggtggtggtggaagtttcc	97.62	g	g	g	t	g	g	t	g	g	t	g	g	a	a	g	t	t	t	c	c
EV-B	46	DQ902713.1	EV-B100	tggtggtggaagttcccgga	100	t	g	g	t	g	g	t	g	g	a	a	g	t	t	c	c	c	g	g	a	gggtggtggtggaagttccc	97.62	g	g	g	t	g	g	t	g	g	t	g	g	a	a	g	t	t	c	c	c
EV-B	47	AY843303.1	EV-B85	tggtggtggaagtttccgga	100	t	g	g	t	g	g	t	g	g	a	a	g	t	t	t	c	c	g	g	a	ggatggtggtggaagtttcc	97.62	g	g	a	t	g	g	t	g	g	t	g	g	a	a	g	t	t	t	c	c
EV-B	48	AB426609.1	EV-B107	tggtggtggaaattccctga	100	t	g	g	t	g	g	t	g	g	a	a	a	t	t	c	c	c	t	g	a	ggatggtggtggaaattccc	97.62	g	g	a	t	g	g	t	g	g	t	g	g	a	a	a	t	t	c	c	c
EV-B	49	KF312882.1	EV-B111	tggtggtggaaatttccaga	100	t	g	g	t	g	g	t	g	g	a	a	a	t	t	t	c	c	a	g	a	ggatggtggtggaaatttcc	97.62	g	g	a	t	g	g	t	g	g	t	g	g	a	a	a	t	t	t	c	c
EV-B	50	AY843304.1	EV-B86	tggtggtggaagtttccaga	100	t	g	g	t	g	g	t	g	g	a	a	g	t	t	t	c	c	a	g	a	ggatggtggtggaagtttcc	97.62	g	g	a	t	g	g	t	g	g	t	g	g	a	a	g	t	t	t	c	c
EV-B	51	AY843305.1	EV-B87	tggtggtggaaatttccgag	95.24	t	g	g	t	g	g	t	g	g	a	a	a	t	t	t	c	c	g	a	g	gggtggtggtggaaatttcc	97.62	g	g	g	t	g	g	t	g	g	t	g	g	a	a	a	t	t	t	c	c
EV-B	52	AY843306.1	EV-B88	tggtggtggaaattccctaa	97.62	t	g	g	t	g	g	t	g	g	a	a	a	t	t	c	c	c	t	a	a	ggttggtggtggaaattccc	97.62	g	g	t	t	g	g	t	g	g	t	g	g	a	a	a	t	t	c	c	c
EV-B	53	AB426608.1	EV-B98	tggtggtggaagttccctaa	97.62	t	g	g	t	g	g	t	g	g	a	a	g	t	t	c	c	c	t	a	a	gggtggtggtggaagttccc	97.62	g	g	g	t	g	g	t	g	g	t	g	g	a	a	g	t	t	c	c	c
EV-B	54	AY843307.1	EV-B97	tggtggtggaagtttcctga	100	t	g	g	t	g	g	t	g	g	a	a	g	t	t	t	c	c	t	g	a	gggtggtggtggaagtttcc	97.62	g	g	g	t	g	g	t	g	g	t	g	g	a	a	g	t	t	t	c	c
EV-B	55	AJ493062.2	EV-B77	tggtggtggaagtttcccga	100	t	g	g	t	g	g	t	g	g	a	a	g	t	t	t	c	c	c	g	a	ggatggtggtggaagtttcc	97.62	g	g	a	t	g	g	t	g	g	t	g	g	a	a	g	t	t	t	c	c
EV-B	56	AY556070.1	EV-B75	tggtggtggaaattcccaga	100	t	g	g	t	g	g	t	g	g	a	a	a	t	t	c	c	c	a	g	a	ggttggtggtggaaattccc	97.62	g	g	t	t	g	g	t	g	g	t	g	g	a	a	a	t	t	c	c	c
EV-B	57	EF127244.1	EV-B93	No data		-	-	-	-	-	-	-	-	-	-	-	-	-	-	-	-	-	-	-	-	--------------------		-	-	-	-	-	-	-	-	-	-	-	-	-	-	-	-	-	-	-	-
EV-B	58	AY208120.2	EV-B78	tggtggtggaaattccctga	100	t	g	g	t	g	g	t	g	g	a	a	a	t	t	c	c	c	t	g	a	gggtggtggtggaaattccc	97.62	g	g	g	t	g	g	t	g	g	t	g	g	a	a	a	t	t	c	c	c
EV-C	1	AF499635.1	CV-A1	tggtggtggaagttaccaga	97.62	t	g	g	t	g	g	t	g	g	a	a	g	t	t	a	c	c	a	g	a	ggatggtggtggaagttacc	100	g	g	a	t	g	g	t	g	g	t	g	g	a	a	g	t	t	a	c	c
EV-C	2	AF499643.1	CV-A22	tggtggtggaagttacccga	97.62	t	g	g	t	g	g	t	g	g	a	a	g	t	t	a	c	c	c	g	a	gggtggtggtggaagttacc	100	g	g	g	t	g	g	t	g	g	t	g	g	a	a	g	t	t	a	c	c
EV-C	3	AF499641.1	CV-A19	tggtggtggaagttacctga	97.62	t	g	g	t	g	g	t	g	g	a	a	g	t	t	a	c	c	t	g	a	ggctggtggtggaagttacc	100	g	g	c	t	g	g	t	g	g	t	g	g	a	a	g	t	t	a	c	c
EV-C	4	EF015886.1	EV-C96	tggtggtggaagttaccgga	97.62	t	g	g	t	g	g	t	g	g	a	a	g	t	t	a	c	c	g	g	a	ggctggtggtggaagttacc	100	g	g	c	t	g	g	t	g	g	t	g	g	a	a	g	t	t	a	c	c
EV-C	5	EU840733.1	EV-C104	tggcggtggaagctacccga	83.33	t	g	g	c	g	g	t	g	g	a	a	g	c	t	a	c	c	c	g	a	ggctggcggtggaagctacc	91.67	g	g	c	t	g	g	c	g	g	t	g	g	a	a	g	c	t	a	c	c
EV-C	6	JQ446368.1	EV-C	tggtggtggaagctaccaga	95.24	t	g	g	t	g	g	t	g	g	a	a	g	c	t	a	c	c	a	g	a	gggtggtggtggaagctacc	97.62	g	g	g	t	g	g	t	g	g	t	g	g	a	a	g	c	t	a	c	c
EV-C	7	MH229997.1	EV-C105	tggtggtggaagctccccga	97.62	t	g	g	t	g	g	t	g	g	a	a	g	c	t	c	c	c	c	g	a	ggatggtggtggaagctccc	95.24	g	g	a	t	g	g	t	g	g	t	g	g	a	a	g	c	t	c	c	c
EV-C	8	GQ865517.1	EV-C109	tggtggtggaagcttcccga	97.62	t	g	g	t	g	g	t	g	g	a	a	g	c	t	t	c	c	c	g	a	ggctggtggtggaagcttcc	95.24	g	g	c	t	g	g	t	g	g	t	g	g	a	a	g	c	t	t	c	c
EV-C	9	JX961708.1	EV-C118	tggtggtggaaactaccgga	95.24	t	g	g	t	g	g	t	g	g	a	a	a	c	t	a	c	c	g	g	a	ggctggtggtggaaactacc	97.62	g	g	c	t	g	g	t	g	g	t	g	g	a	a	a	c	t	a	c	c
EV-C	10	AF499636.1	CV-A11	tggtggtggaaactaccgga	95.24	t	g	g	t	g	g	t	g	g	a	a	a	c	t	a	c	c	g	g	a	ggatggtggtggaaactacc	97.62	g	g	a	t	g	g	t	g	g	t	g	g	a	a	a	c	t	a	c	c
EV-C	11	AF499639.1	CV-A17	tggtggtggaagttaccaga	97.62	t	g	g	t	g	g	t	g	g	a	a	g	t	t	a	c	c	a	g	a	gggtggtggtggaagttacc	100	g	g	g	t	g	g	t	g	g	t	g	g	a	a	g	t	t	a	c	c
EV-C	12	AF499642.1	CV-A20	tggtggtggaaattaccaga	97.62	t	g	g	t	g	g	t	g	g	a	a	a	t	t	a	c	c	a	g	a	ggatggtggtggaaattacc	100	g	g	a	t	g	g	t	g	g	t	g	g	a	a	a	t	t	a	c	c
EV-C	13	AY560657.1	PV-1	tggtggtggaagttgcctga	97.62	t	g	g	t	g	g	t	g	g	a	a	g	t	t	g	c	c	t	g	a	gggtggtggtggaagttgcc	97.62	g	g	g	t	g	g	t	g	g	t	g	g	a	a	g	t	t	g	c	c
EV-C	14	K01392.1	PV-3	tggtggtggaagttacctga	97.62	t	g	g	t	g	g	t	g	g	a	a	g	t	t	a	c	c	t	g	a	ggctggtggtggaagttacc	100	g	g	c	t	g	g	t	g	g	t	g	g	a	a	g	t	t	a	c	c
EV-C	15	M12197.1	PV-2	tggtggtggaaactgcctga	95.24	t	g	g	t	g	g	t	g	g	a	a	a	c	t	g	c	c	t	g	a	gggtggtggtggaaactgcc	95.24	g	g	g	t	g	g	t	g	g	t	g	g	a	a	a	c	t	g	c	c
EV-C	16	EF555645.1	EV-C102	tggtggtggaagctaccaga	95.24	t	g	g	t	g	g	t	g	g	a	a	g	c	t	a	c	c	a	g	a	ggatggtggtggaagctacc	97.62	g	g	a	t	g	g	t	g	g	t	g	g	a	a	g	c	t	a	c	c
EV-C	17	AF499637.1	CV-A13	tggtggtggaaactgcccga	95.24	t	g	g	t	g	g	t	g	g	a	a	a	c	t	g	c	c	c	g	a	ggatggtggtggaaactgcc	95.24	g	g	a	t	g	g	t	g	g	t	g	g	a	a	a	c	t	g	c	c
EV-C	18	AF546702.1	CV-A21	tggtggtggaagttaccaga	97.62	t	g	g	t	g	g	t	g	g	a	a	g	t	t	a	c	c	a	g	a	ggatggtggtggaagttacc	100	g	g	a	t	g	g	t	g	g	t	g	g	a	a	g	t	t	a	c	c
EV-C	19	D90457.1	CV-A24	tggtggtggaagctaccaga	95.24	t	g	g	t	g	g	t	g	g	a	a	g	c	t	a	c	c	a	g	a	ggatggtggtggaagctacc	97.62	g	g	a	t	g	g	t	g	g	t	g	g	a	a	g	c	t	a	c	c
EV-C	20	EF555644.1	EV-C99	tggtggtggaagttgccgga	97.62	t	g	g	t	g	g	t	g	g	a	a	g	t	t	g	c	c	g	g	a	ggttggtggtggaagttgcc	97.62	g	g	t	t	g	g	t	g	g	t	g	g	a	a	g	t	t	g	c	c
EV-D	1	AY426531.1	EV-D68	tggtggtggaaactacctga	95.24	t	g	g	t	g	g	t	g	g	a	a	a	c	t	a	c	c	t	g	a	ggatggtggtggaaactacc	97.62	g	g	a	t	g	g	t	g	g	t	g	g	a	a	a	c	t	a	c	c
EV-D	2	D00820.1	EV-D70	tggtggtggaagctccctga	97.62	t	g	g	t	g	g	t	g	g	a	a	g	c	t	c	c	c	t	g	a	gggtggtggtggaagctccc	95.24	g	g	g	t	g	g	t	g	g	t	g	g	a	a	g	c	t	c	c	c
EV-D	3	DQ916376.1	EV-D94	tggtggtggaaattacccga	97.62	t	g	g	t	g	g	t	g	g	a	a	a	t	t	a	c	c	c	g	a	gggtggtggtggaaattacc	100	g	g	g	t	g	g	t	g	g	t	g	g	a	a	a	t	t	a	c	c
EV-D	4	MK032896.1	EV-D111	tggtggtggaaattaccaga	97.62	t	g	g	t	g	g	t	g	g	a	a	a	t	t	a	c	c	a	g	a	gggtggtggtggaaattacc	100	g	g	g	t	g	g	t	g	g	t	g	g	a	a	a	t	t	a	c	c
