# Supplementary material for: Enterovirus Testing in Hand, Foot, and Mouth Disease and Herpangina: A Highly Sensitive Single-Round VP4–VP2 Reverse-Transcription Polymerase Chain Reaction Assay with a Redesigned Reverse Primer
Source: Viruses. 2026 Apr 30;18(5):527. doi: 10.3390/v18050527 (PMC13211326; doi:10.3390/v18050527)
Supplement: Supplementary file 1 [file viruses-18-00527-s001.zip › Supplementary Table S3.pdf]

Supplementary Table S3. VP4 amino acid identity of representative clinical specimens

Virus type	Specimen ID	Closest reference strain	VP4 length (aa)	p-distance	AA identity (%)	Interpretation
EV-A71	FR-61	EV-A71 Nagoya	146	0	100	Clearly assigned
EV-A71	FR-62	EV-A71 Nagoya	146	0	100	Clearly assigned
EV-A71	FR-58	EV-A71 Nagoya	146	0	100	Clearly assigned
CVA10	FR-28	CV-A10 Kowalki	146	0.0205	97.9	Clearly assigned
CVA10	FR-36	CV-A10 Kowalki	146	0.0205	97.9	Clearly assigned
CVA16	FR-42	CV-A16 G-10	146	0.0205	97.9	Clearly assigned
CVA16	FR-8	CV-A16 G-10	146	0.0205	97.9	Clearly assigned
CVA6	FR-5	CV-A6 Fleetwood	146	0.1369	86.3	Low identity
CVA6	FR-39	CV-A6 Fleetwood	146	0.1369	86.3	Low identity
CVA6	FR-68	CV-A6 Fleetwood	146	0.1369	86.3	Low identity

Notes

Amino acid identity (%) was calculated as **100 × (1 − p-distance)**.

p-distance values were obtained using MEGA X (p-distance model, complete deletion, 146 aa).

All 43 clinical specimens were analyzed; representative specimens illustrating stable assignment and lower-identity cases are shown here.
